# Extreme environmental adaptation mechanisms of Antarctic bryophytes are mainly the activation of antioxidants, secondary metabolites and photosynthetic pathways

**DOI:** 10.1186/s12870-023-04366-w

**Published:** 2023-08-22

**Authors:** Liping Zhang, Zhi Zhang, Junhan Cao, Kai Wang, Ling Qin, Yongjun Sun, Wenming Ju, Changfeng Qu, Jinlai Miao

**Affiliations:** 1 Department of Special Medicine, School of Basic Medicine, Qingdao University, Qingdao, 266071 China; 2 Key Laboratory of Marine Eco-Environmental Science and Technology, First Institute of Oceanography, Ministry of Natural Resources, Qingdao, 266061 China; 3 Homey Group Co.,Ltd, Rongcheng, 264300 China; 4 Laboratory for Marine Drugs and Bioproducts, Qingdao Pilot National Laboratory for Marine Science and Technology, Qingdao, 266237 China; 5 Marine Natural Products R&D Laboratory, Qingdao Key Laboratory, Qingdao, 266061 China

**Keywords:** Antarctica, *Pohlia nutans* M211, Third-generation sequencing, UVB, Low-temperature

## Abstract

The environment in Antarctica is characterized by low temperature, intense UVB and few vegetation types. The *Pohlia nutans* M211 are bryophytes, which are the primary plants in Antarctica and can thrive well in the Antarctic harsh environment. The transcriptional profiling of *Pohlia nutans* M211 under low temperature and high UVB conditions was analyzed to explore their polar adaptation mechanism in the extreme Antarctic environment by third-generation sequencing and second-generation sequencing. In comparison to earlier second-generation sequencing techniques, a total of 43,101 non-redundant transcripts and 10,532 lncRNA transcripts were obtained, which were longer and more accurate. The analysis results of GO, KEGG, AS (alternative splicing), and WGCNA (weighted gene co-expression network analysis) of DEGs (differentially expressed genes), combined with the biochemical kits revealed that antioxidant, secondary metabolites pathways and photosynthesis were the key adaptive pathways for *Pohlia nutans* M211 to the Antarctic extreme environment. Furthermore, the low temperature and strong UVB are closely linked for the first time by the gene HY5 (hlongated hypocotyl 5) to form a protein interaction network through the PPI (protein–protein interaction networks) analysis method. The UVR8 module, photosynthetic module, secondary metabolites synthesis module, and temperature response module were the key components of the PPI network. In conclusion, this study will help to further explore the polar adaptation mechanism of Antarctic plants represented by bryophytes and to enrich the polar gene resources.

**Supplementary Information:**

The online version contains supplementary material available at 10.1186/s12870-023-04366-w.

## Introduction

The growth of plants is restricted in the Antarctic environment due to its lack of water, cold, high salt, and intense ultraviolet (UV) radiation. The bryophyte is one of the few terrestrial vegetation types in the Antarctic continent, which has existed on land for more than 400 million years and is considered the ancestor of land plants [1]. Bryophytes play an indispensable role in the survival of Antarctic microorganisms and invertebrates through the complex food network and are closely related to the circulation of the Antarctic land and sea. They regulate carbon and nitrogen fixation and biological degradation, etc. [2, 3]. Therefore, the reason bryophytes can adapt to the extreme environment in Antarctica must be that they have formed a unique and effective mechanism to cope with biotic and abiotic stress in the long-term evolutionary process.

Although UVA (315–400 nm) and UVB (280–315 nm) are partially absorbed after being absorbed by the ozone layer, the amount that reaches the earth is still very large. So UVB is easily absorbed by biomolecules, causing reactive oxygen species (ROS) to attack protein and DNA structure, resulting in biological function damage. UVB has increased obviously due to the depletion and hole of the ozone layer in Antarctica since 1980 [4]. Though the Antarctic ozone hole reached its peak value of about 24 × 10^6^ km^2^ in early October 2020, UVB irradiance will decrease by up to 40% in the spring of 2100 as the ozone layer continues to recover. However, it was reported that the area of the Antarctic ozone hole was higher than the average of the past decade, and the recovery of Antarctic ozone to pro-1980 levels may be significantly delayed [5]. Antarctic plant self-protection mechanisms have been continuously improved in three aspects, including physical avoidance, synthesis of sunscreen substances, and DNA light repair substances. The rapid response of bryophytes establishes potential UVB protection to increase the synthesis of various antioxidants and secondary metabolites under low doses of UVB stress, which is mainly similar to that of vascular plants [6, 7]. Studies have shown that the bryophytes lineage is the earliest embryonic plant to be exposed to terrestrial UVB radiation [8], so mosses (*Physcomitrella patens*) are more resistant to UVB stress than higher plants (*Arabidopsis thalian*) [9].

UVR8 (UV resistance locus 8) is the only discovered specific receptor that can receive UVB signals to regulate growth and development in plants. The amino acid sequences encoded by green algae, mosses, and other plants are very similar to those of angiosperms with conservative amino acid sequences [8, 10]. Therefore, it is speculated that the function and sequence of UVR8 are conservative in the evolution process over hundreds of millions of years. In response to UVB signals, the HY5 gene produces light morphological reactions and downstream secondary metabolites, such as inhibiting hypocotyl elongation and anthocyanin accumulation [11]. UVR8 plays an indispensable role in the resistance of mosses to UVB stress, because the different reflection patterns of UVR8 may also determine the accumulation of compounds related to the UVB reaction in plants [7].

The adaptability to the low-temperature environment of plans in the Antarctic continent is mainly attributed to their resistance to rapid cell dehydration caused by drought [12]. Some studies also speculate that the survival of Antarctic mosses in this cold environment may be dependent on their ability to maximize photosynthesis in a short period in the summer and minimize respiratory carbon loss under cold conditions [13]. The contents of extracellular soluble sugar are increased with the decrease in temperature [14], improving the cold resistance of plants. Low temperatures cause plants to produce more oxygen free radicals, which destroy the membrane structure, damage plant tissues, and cause metabolic disorders [15]. The active substances (flavonoids, terpenoids, and polyphenols) can scavenge free radicals in time, improving plants' antioxidant capacity in low-temperature environments. UVB and low temperature can be closely linked through the core gene HY5 [16, 17]. UVR8 can negatively regulate the E3 ubiquitin lipase activity of the COP1-SPAs complex to reduce the degradation of HY5. The HY5 regulates the synthesis of hormones, soluble sugars, polyphenols, flavonoids, terpenes, and other substances in plants after receiving the UVB signal to further improve their cold resistance [18]. So, the HY5 is chosen as the core gene to explore the adaptation mechanism of bryophytes to extreme environments.

The *Pohlia nutans* belongs to a species of Pohlia (Bryophyta, Mniaceae) and is mainly distributed in Xinjiang (China), however, the *Pohlia nutans* M211 was obtained on land in Antarctica in 2014. Although the second-generation transcriptome of *Pohlia nutans* has been sequenced, the full-length transcriptome of *Pohlia nutans* M211 was based on the third-generation sequencing platform of PacBio Sequel. The complete gene containing the 5', 3'UTR and polyA tail can be directly obtained without destroying the splicing sequence. Simultaneously, second-generation sequencing data can be used to analyze the transcription-specific expression and obtain more comprehensive annotation information. Therefore, we gained the full-length transcriptomes of *Pohlia nutans* M211 under different levels of low-temperature and UVB stress by a combination of third-generation and second-generation sequencing. Important core genes were explored in depth by WGDNA, AS, and PPI analysis methods, which laid a foundation for further exploring the gene resources of Antarctic bryophytes and obtaining their polar adaptation mechanism.

## Results

### Analysis of transcriptome data

To obtain the gene regulation mechanism of *Pohlia nutans* M211 in Antarctic extreme environment, the RNA sequence was sequenced on the PacBio Sequel platform and a large number of long reads were shown in Table S1. The SMRT (Single-Molecular Real-Time) sequencing method produced 1,074,872 polymerase reads (Mean length: 53,947, and N50: 111,535) and 29,661,756 subreads (Mean length: 1881, and N50: 2247). Then 1,074,872 CCS (circular consensus sequence, mean length: 2409: N50 2584) were obtained by self-correction of subreads sequence, 831,751 FLNCS (Full-length nonchimera, mean length: 2204, N50: 2409) were identified by detecting whether CCS contains 5'-primer, 3'-primer and polyA. Finally, cluster consensus was obtained by clustering the FLNC sequence of the same transcript with a hierarchical n*log(n) algorithm and the polished consensus was achieved by correcting the cluster consensus sequence with arrow software. The 78,059 polished consensus sequences (Mean length: 2217, N50: 2431) were obtained by LoRDEC software using Illumina sequencing data (Table S2) for subsequent analysis. The CD-HIT software was used to cluster the corrected transcript sequences through sequence alignment, and 43,101 transcripts (Mean length: 2,268, N50: 2,487) after redundancy were got according to the 95% similarity between the sequences.

### Transcription factors, lncRNA, and gene expression correlation analysis

Transcription factor (TF) is a group of protein molecules that can specifically bind to the specific sequence upstream of the 5 'end of a gene, so as to ensure that the target gene is expressed. The top 10 types of predicted transcription factors were predicted by iTAK software as followed, AP2/ERF-ERF (186), BHLH (169), C3H (137), C2C2-dof (134), bZIP (126), MYB—related (111), B3 (95), GARP-G2-like (93), NAC (93) and GRAS (87) (Figure S1a). The specific up-regulation and down-regulation of TFs (AP2/ERF, WRKY, bHLH, bZIP, MYB, NAC, and C2H2) under different experimental conditions were shown in Figure S1b-f.

**Fig. 1:**
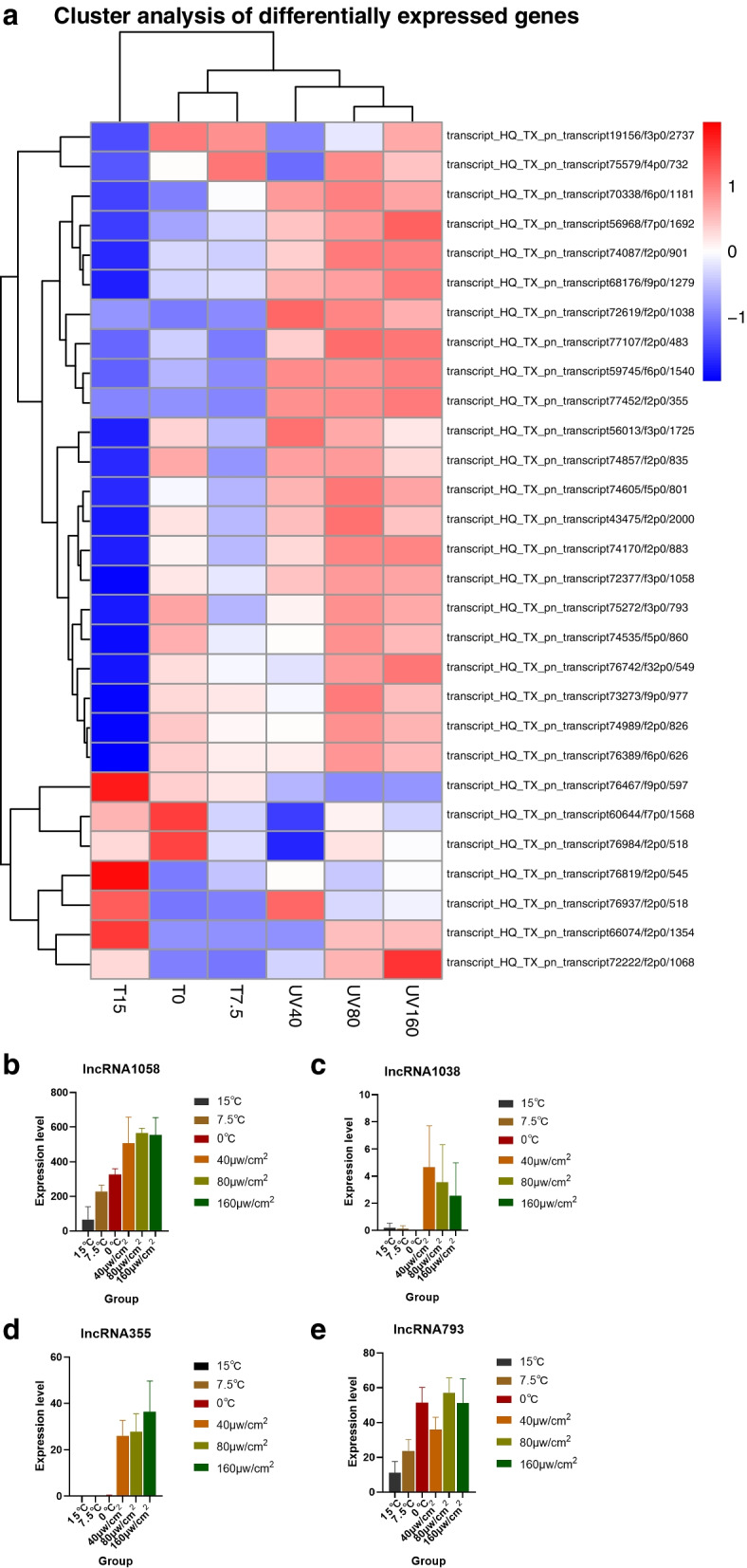
The lncRNA expression under differential treatments. (The a is the hierarchical clustering heat map of differential lncRNA expression, where red represents highly expressed genes and blue represents lowly expressed genes. The b-e are expression levels of lncRNA1058, lncRNA1038, lncRNA355 and lncRNA793 respectively under different conditions, with different colors representing different experimental treatments. Results were expressed as the mean ± SD, *n* = 3)

The lncRNA (long-chain non-coding RNA) is a kind of RNA molecule that has a transcript length of more than 200nt but does not code for proteins. The length distribution density map of the predicted lncRNA was predicted through CNCI, PLEK, CPC2 software, and the Pfam database (Figure S2a), and 10,532 lncRNA sequences were ultimately acquired. The clustering heat map of the differential lncRNA expression can be used to determine the expression of lncRNA under various treatment settings, as illustrated in Fig. 1a. It was crucial that 4 lncRNAs were found under low-temperature and UVB conditions to regulate metabolism, photosynthesis, and UVB radiation injury repair, respectively (Fig. 1b-e), which were of great significance for *Pohlia nutans* M211 to adapt to the harsh environment in Antarctica. The transcript_HQ_TX_pn_transcript72377/f3p0/1058 (lncRNA1058) can participate in regulating adenylate kinase ( ADK), which is associated with plant metabolism and is found in many organisms to help maintain energy homeostasis [19]. The transcript_HQ_TX_pn_transcript72619/f2p0/1038 (lncRNA1038) and transcript_HQ_TX_pn_transcript77452/f2p0/355 (lncRNA355) regulate photosystem II protein D2 (PSBD) and photosystem II N protein (PSBN), both of which belong to photosystem II protein (PSII). PSII is a membrane protein complex that catalyzes light-induced water oxidation in oxygen-containing photosynthesis and performs the initial reaction of photosynthesis in higher plants, algae, and cyanobacteria [20]. Compared with no UVB treatment, UVB stress enhanced the photosynthetic capacity of *Pohlia nutans* M211. The transcript_HQ_TX_pn_transcript75272/f3p0/793 (lncRNA793) helps to regulate the stress-induced protein UVI31 + , which is activated by UV radiation and is involved in DNA repair [21]. As a result, lncRNAs play an important role in various biological processes of plant development in harsh environments.

The correlation coefficients within and between groups of samples were calculated according to the FPKM values of all genes in each sample in Figure S2b. It was considered that this experiment had good repeatability and reasonable experimental treatment because of R^2^ > 0.8 in each treatment group [22].

### Identification and cluster analysis of DEGs

The differential genes of all groups were combined as a volcano map in Figure S3a-e. And 12,751 (0 ℃ vs 15 ℃), 10,069 (7.5 ℃ vs 15), 4136 (UVB 40 μW/cm^2^ vs 0 μW/cm^2^), and 4069 (UVB 80 μW/cm^2^ vs 0 μW/cm^2^), 2649 (UVB160 μW/cm^2^ vs 0 μW/cm^2^) DEGs were identified by BH method with |log_2_ (Fold Change)|> 1 & Padj < 0.005. The overlap of differential genes among various comparison combinations can be seen using a Venn diagram, and some common or distinctive differential genes of comparison combinations can be screened out. As shown in the Figure S4a, a total of 1,460 differential genes (0 ℃ vs 7.5 ℃) and 2220 differential genes (0 ℃ vs 15 ℃) were found, so the variation degree of differential genes increased with the temperature. Compared with the differential genes of middle UVB treatment (80 μW/cm^2^ VS 0 μW/cm^2^, 1375) and high UVB treatment (160 μW/cm^2^ VS 0 μW/cm^2^, 1456), it was found that low UVB radiation could induce more gene changes (UVB 40 μW/cm^2^ VS 0 μW/cm^2^, 1461) in Figure S4b.

**Fig. 2:**
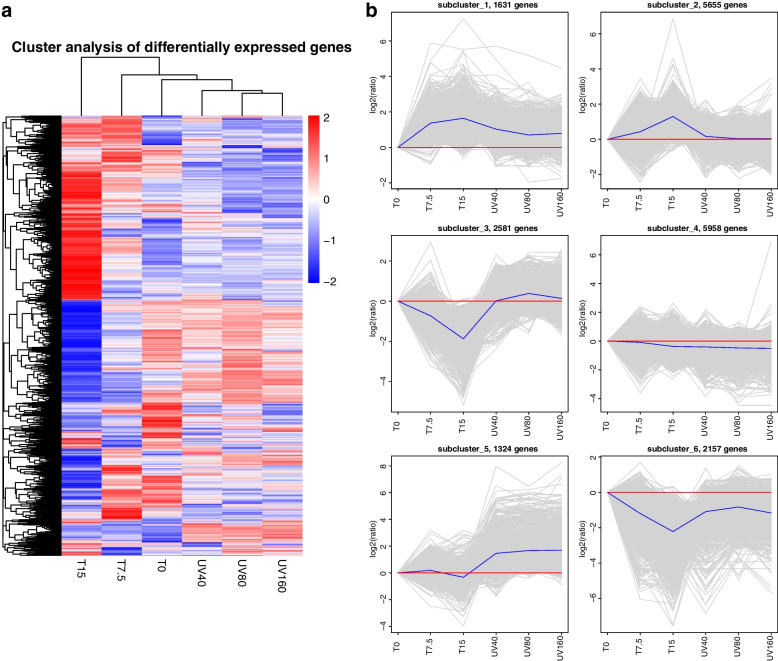
The overall FPKM hierarchical clustering map and cluster clustering result graph. (Where log_10_ (FPKM + 1) values are normalized transformed (scale number) and clustered, with red indicating highly expressed genes and blue indicating lowly expressed genes, and the color ranges from red to blue indicating log_10_ (FPKM + 1) from high to low. The cluster clustering result graph, where the gray lines in each subgraph represent the relative expression levels of genes in a cluster under different experimental conditions (taking the expression level of the first sample as the benchmark, as shown by the red lines), and the blue lines represent the average relative expression levels of all genes in this cluster under different experimental conditions)

The 19,306 genes with similar expression patterns were clustered into FPKM hierarchical clustering map by clustering analysis of different genes, which changed significantly after low-temperature and UVB treatment (Fig. 2a). Next, we clustered the relative expression levels log_2_ (ratios) of different genes by the H-cluster method and divided the different genes into 6 categories by different clustering algorithms (Fig. 2b). There were 724 differential genes related to the metabolic process, 671 differential genes related to the oxidation process, and 30 differential genes related to the fatty acid biosynthesis process among those in cluster 1 (1631, Additional file 1), which share a temperature change pattern with cluster 2 (5655, Additional file 2) and exhibit a positive correlation with temperature. The temperature patterns of cluster 3 (2581, Additional file 3) and cluster 6 (2157, Additional file 6) both showed a downward pattern with increasing temperature, among which there were 253 differential genes related to the metabolic process, 534 differential genes related to the metabolic process, and 54 differential genes related to the fatty acid biosynthetic process. Cluster 4 (5958, Additional file 4) and cluster 5 (1324, Additional file 5) were positively and negatively correlated with the intensity of UVB respectively, among which there were 485 genes related to oxidoreductase and 94 genes related to DNA repair.

### Differential gene KEGG analysis

**Fig. 3:**
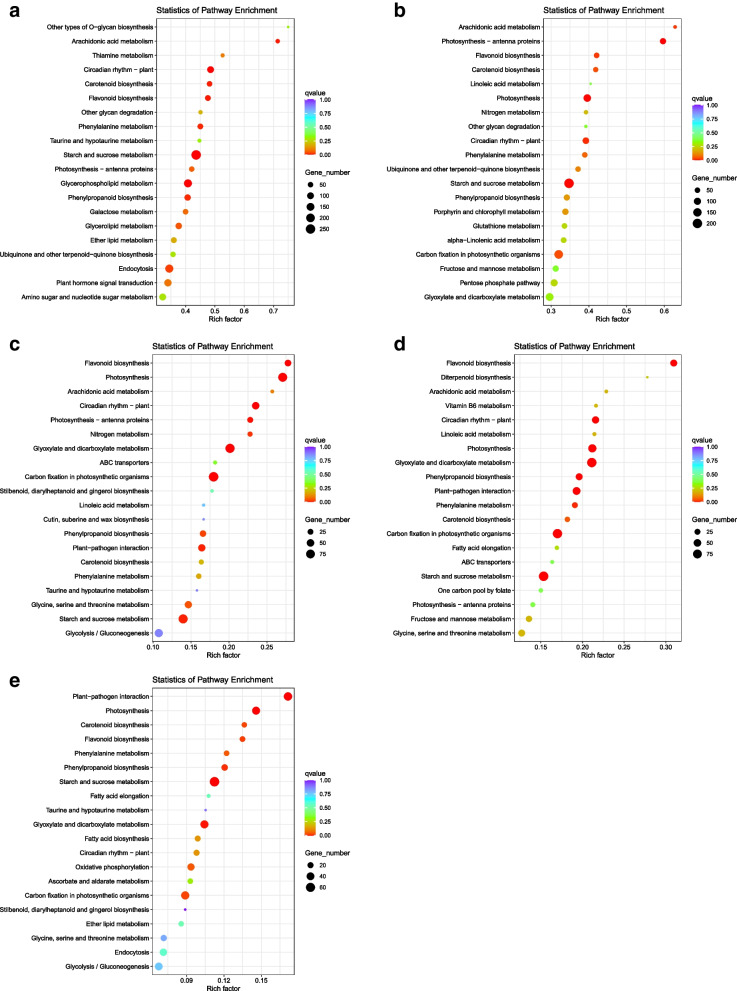
The KEGG enrichment scatter diagram of differential genes. (The horizontal axis represents the rich factor, the size of the dot represents the number of differentially expressed genes in this pathway, and the color of the dot corresponds to different value ranges. The a and b are 0 ℃, and 7.5 ℃ compared with 15 ℃, respectively. The d, e, and f are 40 μW/cm^2^, 80 μW/cm^2^, and 160 μW/cm^2^ compared with 0 μW/cm.^2^ respectively)

The scatter diagram is a graphical display of KEGG enrichment analysis results, which are measured by the rich factor, value, and the number of genes enriched. We selected the 20 pathway entries with the most significant enrichment to display in the diagram (Fig. 3a-e). The main enrichment-related pathways of DEGs under low-temperature treatment were photosynthesis, phenylpropanoid biosynthesis, carotenoid biosynthesis, arachidonic acid metabolism, flavonoid biosynthesis, fructose and mannose metabolism, porphyrin and chlorophyll metabolism and ubiquinone and other terpenoid quinone biosynthesis, etc. Those under UVB treatment were flavonoid biosynthesis, photosynthesis, arachidonic acid metabolism, taurine and hypotaurine metabolism, phenylalanine metabolism, carotenoid biosynthesis, glycine, serine, and threonine metabolism, fructose and mannose metabolism, base excision repair, stilbenoid, diarylheptanoid and gingerol biosynthesis, plant hormone signal transduction, vitamin b6 metabolism, and steroid biosynthesis, etc.

### WGCNA analysis

**Fig. 4:**
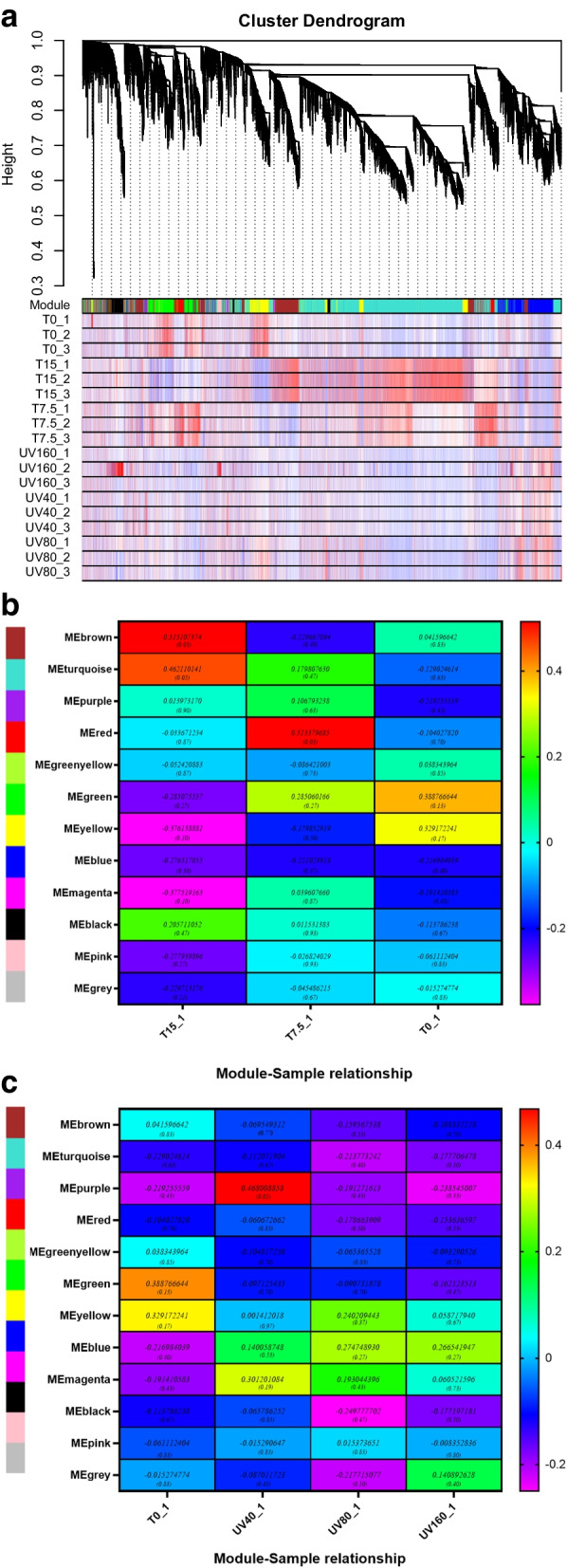
The gene co-expression network gene clustering number and heat map of association under different conditions. (Each row and column represent a module, with darker colors (red or green) indicating stronger correlations, and lighter squares indicating weaker correlations)

WGCNA is a commonly systematic method to study the construction of gene co-expression networks, which is widely used in the international biomedical field for mining core genes from multi-sample transcriptome data. To investigate the *Pohlia nutans* M211 adaption mechanism in the polar environment, a total of 43,104 genes were screened by co-expressions with the soft threshold of β = 5, which were similar to greater than 75% of the WGCNA (Figure S5a, b). The final structures were combined and constructed 12 co-expression modules that were black module (number of genes, 907), blue module (number of genes, 2673), brown module (number of genes, 2554), green module (number of genes, 1905), yellow module (number of genes, 78), grey module (number of genes, 283), magenta module (number of genes, 159), pink module (number of genes, 195), the purple module (number of genes, 115), red module (number of genes, 1094), turquoise module (number of genes, 9448) and yellow module (number of genes, 2113), among which turquoise, brown and blue modules have more than 2500 genes (Fig. 4a and Figure S6a-c). They were merged and fused according to the correlation of 75% in the modules obtained in the previous step. In combination with the different conditions of samples (0 ℃, 7.5 ℃, 15 ℃, 0 μW/cm^2^, 40 μW/cm^2^, 80 μW/cm^2^, and 160 μW/cm^2^), which were represented by different colors in Fig. 4b, c. The modules MEbrown and MEturqoise showed a significant positive correlation at 15 ℃ (*r* = 0.5151, *P* < 0.05; *r* = 0.462, *p* = 0.05), and MEpurple module under the condition of 40 μW/cm^2^ was a significantly positive correlation (*r* = 0.4680, *P* = 0.05), therefore we performed in-depth mining of the three modules.

**Fig. 5:**
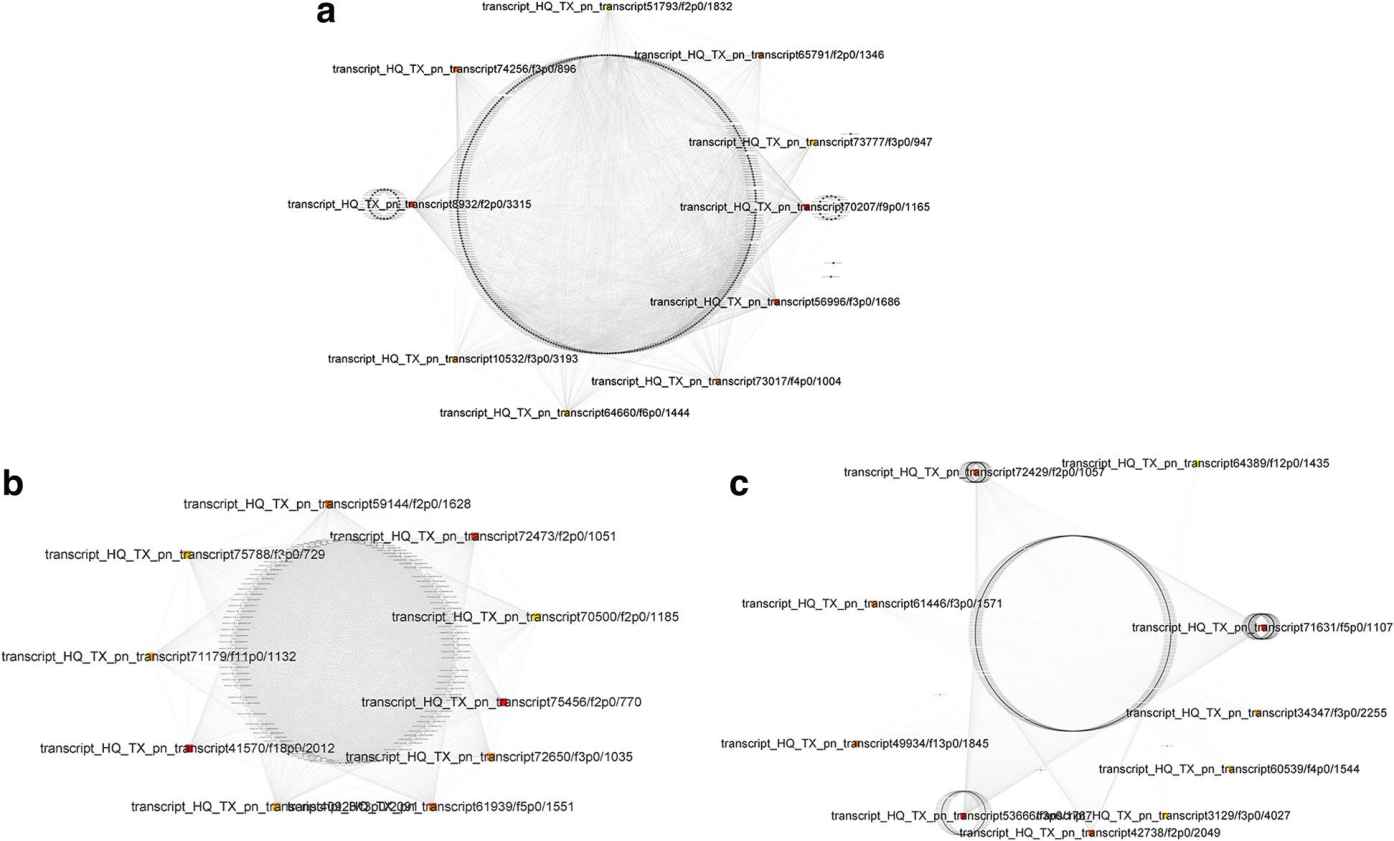
Gene co-expression network of hub genes in MEbrown (**a**), MEturqoise (**b**), and MEpurple (**c**) modules. (the colored squares represent the selected core genes)

The low-temperature related GO pathways of MEbrown and MEturqoise were shown in Table 1, and the pathways related to antioxidants were GO: 0055114 oxidation–reduction process, related to secondary metabolites were GO: 0016114 terpenoid biosynthetic process, GO: 0006694 steroid biosynthetic process, GO: 0005992 trehalose biosynthetic process and GO: 0006000 fructose metabolic process, related to DNA repair were GO: 0006310 DNA repair and GO: 0006310 DNA recombination, related to photosynthesis were GO: 0010207 photosystem II assembly and GO: 0015979 photosynthesis, in addition, related to ion transport were GO: 0006814 sodium ion transport and GO: 0006813 potassium ion transport. Table 2 displays the GO pathways of MEpurple that are related to UVB, these pathways include the gene repair pathway (GO: 0006281 DNA repair), lipid metabolism (GO: 0006629 lipid metabolic process), antioxidant pathway (GO: 0055114 oxidation–reduction process), secondary metabolites pathway (GO: 0008299 isoprenoid biosynthetic process), and pathways related to lipid metabolism and oxidative stress (GO: 0006629 lipid metabolic process). The co-expression network diagrams of each module were shown respectively in Fig. 5, and the top core ten genes and their functions were listed in Table S4. The ten top core genes selected by the degree in MEbrown and MEturqoise modules were closely related to antioxidant and ion transport through the comparison of the NCBI database. Most of the top ten core genes of the MEpurple module screened by the above strategy were related to drying. UVB radiation regulates stomatal behavior and affects water metabolism of plants by affecting signal molecules such as H_2_O_2_, Ca^2+^, ABA and NO. Although there has been limited research on drought response under UVB stress, studies have shown that UVB radiation can reduce water content of plants [23]. So, these results provided insight into the mechanism of plant resistance to UVB.

**Table 1:** The description of the pathway related to low-temperature tolerance in the MEbrown and MEturqoise modules

Module type	Biological Pathway Description	Gene Ontology Biological Pathway	Number of genes
MEbrown	response to stress	GO: 0006950	20
	metabolic process	GO: 0008152	274
	terpenoid biosynthetic process	GO: 0016114	8
	oxidation–reduction process	GO: 0055114	283
	photosynthesis	GO: 0015979	74
	fatty acid metabolic process	GO: 0006631	19
	steroid biosynthetic process	GO: 0006694	26
	DNA repair	GO: 0006281	18
	cell redox homeostasis	GO: 0045454	29
	response to auxin	GO: 0009733	3
	proton transport	GO: 0015992	29
	potassium ion transport	GO: 0006813	20
	biosynthetic process	GO: 0015991	185
	cell redox homeostasis	GO: 0045454	29
	photosystem II assembly	GO: 0010207	5
	trehalose biosynthetic process	GO: 0005992	4
MEturqoise	trehalose biosynthetic process	GO: 0005992	13
	terpenoid biosynthetic process	GO: 0016114	26
	steroid biosynthetic process	GO: 0006694	48
	sodium ion transport	GO: 0006814	14
	secondary metabolic process	GO: 0019748	6
	riboflavin biosynthetic process	GO: 0009231	18
	response to stress	GO: 0006950	46
	response to oxidative stress	GO: 0006979	15
	response to biotic stimulus	GO: 0009607	24
	response to auxin	GO: 0009733	7
	potassium ion transport	GO: 0006813	40
	oxidation–reduction process	GO: 0055114	698
	photosystem II assembly	GO: 0015979	9
	phospholipid biosynthetic process	GO: 0008654	14
	nucleotide-excision repair	GO: 0006289	17
	heme biosynthetic process	GO: 0006783	31
	fructose metabolic process	GO: 0006000	10
	fatty acid biosynthetic process	GO: 0006633	56
	DNA recombination	GO: 0006310	72
	DNA repair	GO: 0006310	86

**Table 2:** The description of pathway related to UVB tolerance in the MEpurple

Biological Pathway Description	Gene Ontology Biological Pathway	Number of genes
DNA repair	GO: 0006281	1
isoprenoid biosynthetic process	GO: 0008299	1
lipid metabolic process	GO: 0006629	3
oxidation–reduction process	GO: 0055114	13
response to stress	GO: 0006950	6

### Alternative splicing analysis of genes

**Fig. 6:**
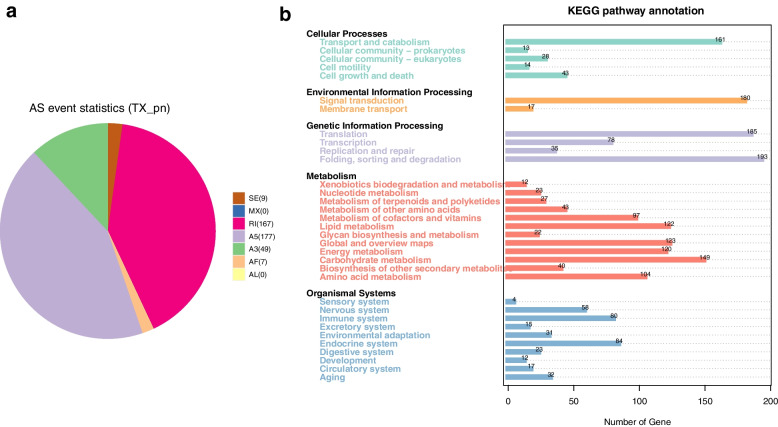
The proportion of AS types (**a**) and KEGG analyses (**b**)

The extensive changes of AS in regulating the biosynthesis of secondary metabolites under the stimulus of harsh environment, is considered to be a method for plants to resist biological and non-biological stresses [24]. The 7 kinds of alternative splicing types with 1538 reconstructed genes were followed in Fig. 6a, skipping exon (SE, accounting for 0.59%), mutually exclusive exons (MX, accounting for 0.0%), retained intron (RI, accounting for 10.86%), alternative 5' splice sites (A5, accounted for 11.51%), alternative 3' splice sites (A3, accounting for 3.19%), alternative first exons (AF, accounting for 0.46%), and alternative last exons (AL, accounting for 0.0%). The pathways enriched to KEGG were divided into five classes (cellular processes, environmental information processing, genetic information processing, metabolic metabolism, and organismal systems). The analysis of KEGG pathways of AS genes was closely related to the Antarctic extreme environment adaptation mechanism of *Pohlia nutans* M211 including replication and repair, lipid metabolism, metabolism of cofactors and vitamins, metabolism of terpenoids and polyketides, amino acid metabolism and environmental adaptation (Fig. 6b). AS can regulate the diversity of its transcriptome and protein to affect the expression of stress-related pathways and code shear genes with the changes in the environment [24]. Therefore, the response of plants to stress is also closely related to the changes of AS in plants.

### Protein–protein interaction networks

**Fig. 7:**
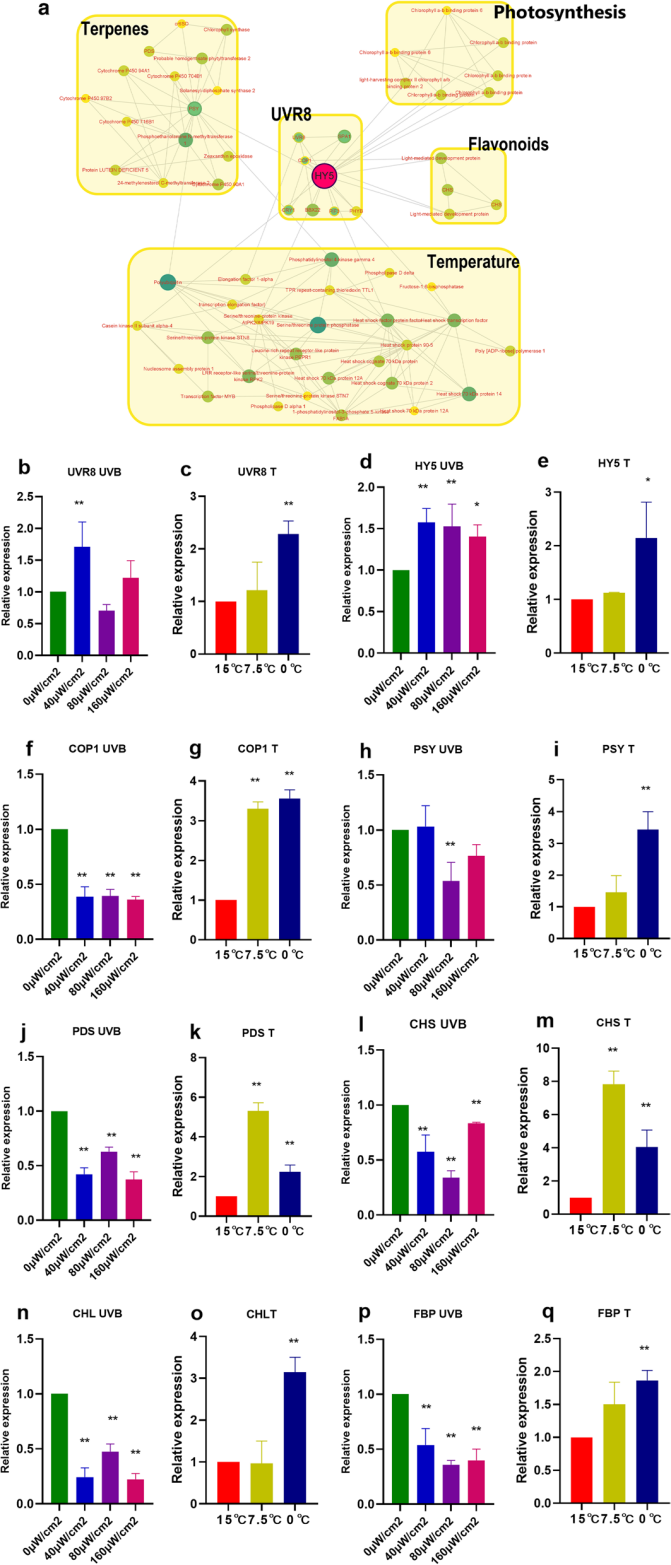
The PPI network with HY5 as the core and relative expression level of key genes. (The name of the measured indicator + UVB represents the change in the gene under UVB conditions, and the name of the measured indicator + T represents the change in the gene under -low-temperature conditions. Results were expressed as the mean ± SD, *n* = 3)

The PPI is a network composed of interacting proteins and conducive to in-depth understanding and excavation of the functional protein. HY5 regulates transcription factors of a large number of genes by directly binding cis-regulatory elements to regulate hormone, nutrition, abiotic stress (abscisic acid, salt, cold, ultraviolet), and reactive oxygen signal pathways at the central position of the transcription network hub [11]. In this study, Cytoscape software (v3.9.0) was used to create a PPI network with 64 nodes and 171 DEGs using 5344 DEGs with HY5 as the central gene. As shown in Fig. 7a, the results showed that the classification of interacting protein with HY5 as the core was mainly related to photosynthesis, temperature, and the synthesis of secondary metabolites (terpenes and flavonoids). The key genes and expression levels in each taxon selected from PPI were shown in Table S6. To verify the accuracy of the transcriptome, we selected the gene similarity with the same function in *Arabidopsis thaliana* and analyzed the mRNA relative expression by RT-qPCR (UVR8, HY5, COP1, PDS, PSY, FBP, CHS, and CHL). As shown in Fig. 7 b-q, the changing trend was similar to that of the transcriptome, suggesting the transcriptome results were credible. In low-temperature, the expression of key genes in PPI interaction modules was up-regulated. Although the expression of secondary metabolites synthesis genes (PSY, PDS, CHS and FBP) was down-regulated after UVB treatment compared with that without UVB treatment, their expression increased with the increase of UVB intensity. In the UVB environment, the expression levels of UVR8 and HY5 increased significantly at 40 μW/cm^2^ UVB, while HY5 increased significantly at 80 μW/cm^2^ and 160 μW/cm^2^ intense UVB. The expression levels of COP1 were extremely significantly decreased under UVB stress, which helped to accumulate HY5 and better-regulated downstream genes to resist UVB and low-temperature environments. Under 40 μW/cm^2^ UVB stress, the expression of one of the three copies of photosynthesis-related genes (phytochrome b, PHYB, and light-harvesting chlorophyll a/b binding protein, LHCB2) increased, which may improve the photosynthesis of moss in the presence of low UVB radiation. The expression of tripeptide-repeat thioredoxin-like 1 (TTL1) increased significantly under UVB treatment, which regulates the transcription level of many dehydration-responsive genes to improve early dehydration.

### The levels of antioxidants, secondary metabolites, and chlorophyll

**Fig. 8:**
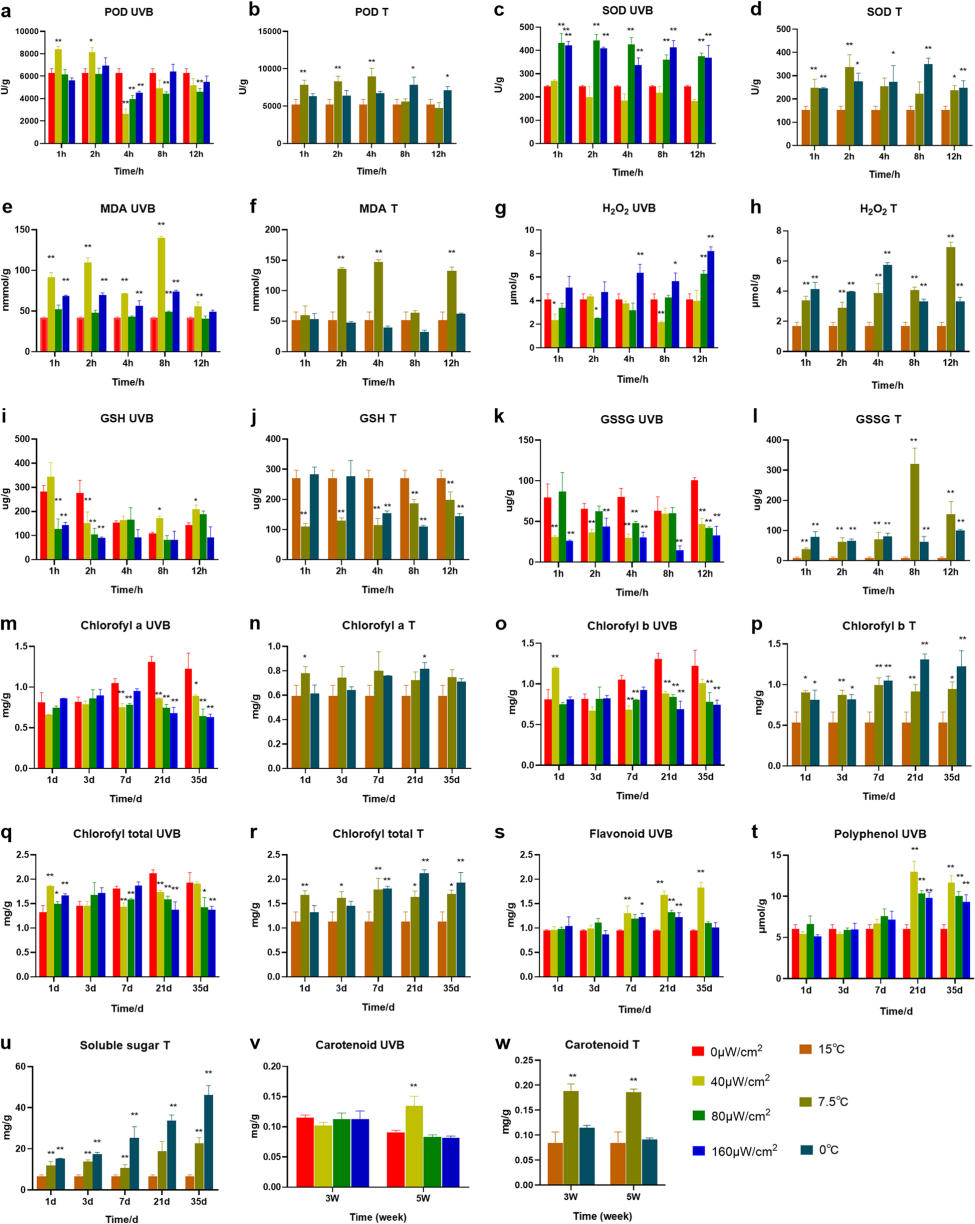
Measurement of levels of antioxidants (**a**-**l**), chlorophyll (**m**-**r**), and secondary metabolites (**s**-**w**). (The name of the measured index + UVB represents that the sample was obtained under UVB conditions, and the name of the measured index + T represents that the sample was obtained under low-temperature conditions. Results were expressed as the mean ± SD, *n* = 3)

In order to further verify that the mechanism of *Pohlia nutans* M211 adapting to low temperature and UVB conditions mainly activates antioxidant activity, stimulates the production of functional metabolites and improves photosynthetic capacity. The levels of antioxidant enzymes (POD, SOD, GSH and GSSG), oxidation products (H_2_O_2_ and MDA), secondary metabolites (flavonoids, polyphenols, soluble sugar, and carotenoid) and chlorophyll (chlorophyll a, chlorophyll b and total chlorophyll) were measured through the method of biochemical kits. POD is a common oxidase in plants and works together with SOD to remove excess free radicals, thus improving the stress resistance of plants. POD activity was significantly increased compared with 0 μW/cm^2^ at 1 h and 2 h when UVB was 40 μW/cm^2^ (*P* < 0.01 and *P* < 0.05), and SOD activity was significantly increased compared with 0 μW/cm^2^ when UVB was 80 μW/cm^2^ and 160 μW/cm^2^ (*P* < 0.01) in Fig. 8a, c. The activity of POD was significantly increased in the first 4 h under low-temperature of 7.5 ℃ (*P* < 0.01), and was significantly increased in the 8th and 12th hours under low-temperature of 0 ℃ (*P* < 0.05) (Fig. 8b). The activity of SOD was significantly increased in the first 2 h and the 12th hour under 7.5 ℃ (*P* < 0.01and *P* < 0.05), and was significantly increased at 0 ℃ (*P* < 0.01 and *P* < 0.05) (Fig. 8d). The aforementioned findings indicated that *Pohlia nutans* M211 could boost the antioxidant enzyme activity to increase the antioxidant capacity at low-temperature and UVB. GSH can reduce H_2_O_2_ produced in cells to H_2_O, while GSH is oxidized to GSSG. When the antioxidant mechanism in plants cannot remove reactive oxygen species in time, too many reactive oxygen species will lead to membrane lipid peroxidation. The MDA is one of the products of membrane lipid peroxidation in plants under adverse conditions. Compared with 0 μW/cm^2^, the GSH level of the 40 μW/cm^2^ group was significantly increased from 8 to 12 h (*P* < 0.05), the GSSG was extremely significantly reduced under different UVB treatments, the H_2_O_2_ content was significantly reduced in the first 2 h and the 8th hour under the 40 μW/cm^2^ condition (*P* < 0.05 and *P* < 0.01), and the MDA content decreased at the 12th hour under UVB treatment of 40 μW/cm^2^ and 160 μW/cm^2^ (Fig. 8e, g, i, k), which indicated that the lipid peroxidation level was relieved with time. However, the content of GSH decreased, and the contents of GSSG, H_2_O_2,_ and MDA also increased at low-temperature (Fig. 8f, h, j, l), which speculated that the antioxidant pathway of glutathione was not used to reduce ROS and alleviate lipid peroxidation at low-temperature, but the low-temperature environment with UVB treatment had certain activation effect on this pathway. Under adverse conditions, plants not only scavenge free radicals by producing antioxidant enzymes but also produce secondary functional metabolites to maintain their redox balance. Flavonoids, polyphenols, and carotenoids can scavenge ROS produced by UVB stimulation through hydroxyl groups or conjugated structures in their structure. The contents of flavonoids and polyphenols were significantly increased after the 7th and 21st days under the UVB condition, respectively (*P* < 0.05 and *P* < 0.01), and carotenoids were significantly increased after the 35th day under the 40 μW/cm^2^ condition (*P* < 0.05) (Fig. 8s, t, v). As a result of the findings, the secondary metabolites gradually accumulated with the prolongation of UVB treatment, which were critical for the survival of *Pohlia nutans* M211 in the UVB environment. The cell membrane of plants will harden due to the difference in low temperature, reducing the permeability and destroying the cell function. Non-polar carotenoids tend to be randomly distributed in the hydrophobic interior of the lipid bilayer to improve the fluidity of the membranes [25]. Additionally, soluble sugars in plants can also reduce cell dehydration by improving the intracellular osmotic pressure. At low-temperature of 7.5 ℃, the content of carotenoids increased significantly after 21 to 35 days (*P* < 0.01), and the content of soluble sugar increased significantly at low-temperature (*P* < 0.01), which indicated that *Pohlia nutans* M211 could improve the freezing resistance of *Pohlia nutans* M211 by increasing the content of carotenoids and soluble sugar in low-temperature environment (Fig. 8u, w). It is well known that photosynthesis is the foundation of plant survival and will be seriously damaged in low-temperature and strong UVB environments. The chlorophyll content is positively correlated with the photosynthetic capacity of plants. The results in Fig. 8, m, o, q showed that chlorophyll b content presented an extremely significant increase on the 1st day under the condition of 40 μW/cm^2^ (*P* < 0.01), and total chlorophyll content significantly increased on the 1st day under the conditions of 40 μW/cm^2^, 80 μW/cm^2^, and 160 μW/cm^2^ (*P* < 0.01and *P* < 0.05). The changes were increased significantly on the 1st and 21st days (*P* < 0.05) at 7.5 and 0 ℃, respectively, and chlorophyll b and total chlorophyll content were significantly increased under low-temperature (*P* < 0.05 and *P* < 0.01) (Fig. 8 n, p, r). The biochemical tests listed above match the transcriptome analysis results. In a word, the reason *Pohlia nutans* M211 has been able to adapt to the harsh Antarctic environment for a long time was closely related to the improvement of its antioxidant system, the production of secondary metabolites and enhancement photosynthesis.

## Discussion

The bryophytes, which are typical terrestrial plants in Antarctica, have aroused great interest in the processes behind their long-term survival and evolution under the harsh conditions of the Antarctic low-temperature and extreme ultraviolet radiation environment. The gametophytes of *Pohlia nutans* M211 were collected and sequenced at low-temperature and UVB treatment. There were 7 transcription factors related to low temperature and UVB adaptation, which were AP2/ERF, WRKY, bHLH, bZIP, MYB, NAC, and C2H2, respectively. The AP2/ERF family factors play important regulatory roles in many biological and physiological processes, such as plant morphogenesis, response mechanisms to various stresses, hormone signal transduction, and metabolite regulation [26]. For example, AP2/ERF is involved in the biosynthesis of sesquiterpene and also regulates the accumulation of carotenoids. The RD29A and COR47 factors are regulated by the WRKY transcription factor, a crucial node in the ABA response signaling network, which has an advantageous impact on drought and stomatal closure [27]. In addition, the WRKY transcription factor can not only scavenge reactive oxygen species but also enhance the adaptability of plants to drought stress and low-temperature stress. MYB and bHLH play a regulatory role in the synthesis of flavonoids, and MYB, bHLH, and NAC are also highly correlated with the content of anthocyanins [28]. C2H2 has also made important progress in the regulation of abiotic stress and has positive mediating effects on salt stress, low-temperature stress, and drought stress [29]. For instance, the expression of antioxidant enzyme genes (ascorbic acid peroxidase 1, Apx 1) can be regulated by C2H2 zinc finger protein under abiotic stress, which can scavenge H_2_O_2_ in plants. Therefore, it was projected that the AP2/ERF, WRKY, bHLH, bZIP, MYB, NAC, and C2H2 would increase survivability in the Antarctic. Hierarchical clustering analysis of differential lncRNA expression levels revealed 1 lncRNA related to low-temperature (ADK), 2 lncRNA related to photosynthesis (PSBD, PSBN), and 1 lncRNA related to ultraviolet radiation (UVI31 +). ATP can maintain cell activity under dehydration stress, and its biosynthesis is related to ADK [30]. Studies have also revealed that some enzymes (ADK and catalase) in tomatoes are stimulated under the special environment of dehydration and drying [31], which indicated that ADK was involved in the stress responses of plants and was the key gene for studying the polar adaptation mechanism of *Pohlia nutans* M211. The PSBD and PSBN genes, which encode the D2 protein of the photosystem II action center, are important in photosynthesis [32]. PSBN is necessary for assembling the reaction center of photosystem II, and its inactivation leads to the strong destruction of photosynthesis [33]. Therefore, improving the photosynthetic capacity under low-temperature and UVB stress is one of the mechanisms of adapting to extreme environments. UVI31 + possesses a common BolA domain and increases the UV resistance of *E. coli* expressing UVI31 + by 1000 times [21]. UVI31 + is activated in a specific response to DNA damage and may play a controlling role in the cell cycle process after DNA damage (ultraviolet radiation) [34]. Accordingly, our transcription found that lncRNA involved in the regulation of UVI31 + was up-regulated under UVB treatment, indicating that UVI31 + played an important role in gene repair under UVB radiation.

Through KEGG analysis, *Pohlia nutans* M211 activated antioxidant pathway, fatty acid synthesis pathway, photosynthesis pathway and secondary metabolite pathways in extreme conditions. Environmental stress will destroy the plant's ability to photosynthesis and change the gene expression of PSII reaction center protein synthesis and D1/D2 reaction center protein and other components [35]. The carotenoids can improve the nutritional composition and production of the plant and are important for the photoprotective action of photosynthesis [36]. Thus, *Pohlia nutans* M211 can enhance photosynthesis by activating the carotenoid synthesis pathway. The activation of flavonoid metabolic pathways can produce flavonoid substances (anthocyanins, flavonoids and chalcones), assisting Pohlia *nutans* M211 to remove oxygen free radicals and improve the repair ability of self-injury under the low-temperature and UVB conditions [37]. The synthesis of fructose and mannose is helpful to improve the osmotic pressure of *Pohlia nutans* M211 in low-temperature environment. The production of unsaturated fatty acids (arachidonic acid and carotenoid) can increase the membrane fluidity of *Pohlia nutans* M211 to reduce low-temperature damage. The DNA in tissues is especially sensitive to damage under UVB conditions. The activation of the base excision repair pathway ensures normal cell metabolism and the stability of genetic material [38]. Therefore, *Pohlia nutans* M211 can adapt to low-temperature and UVB environments by activating antioxidant pathways, improving its photosynthetic capacity, promoting the production of secondary metabolites, and repairing base damage, which was consistent with the results measured by the biochemical kit.

The MEbrown and MEturqoise modules were shown to be related to low temperature by WGCNA analysis, and the top ten core gene functions of these modules were mostly related to antioxidant and ion transport. Under adverse environments, various reactive oxygen species (H_2_O_2_, -OH and O_2_-) attack the cell membrane structure and biomolecules, resulting in metabolic disorder. The activation of the antioxidant function helps to scavenge ROS and achieves the purpose of survival of the plant by directly acting on the oxygen free radicals and indirectly consuming the free radical-producing intermediate substances [39, 40]. Ion transport is the basis of plants' nutrient absorption, mediates the circulation of various ions in plants, and is the precursor of the Ca^2+^ signaling pathway, which ultimately participates in antifreeze pathways in plants [41]. Most of the top ten core genes screened by the MEpurple module in UVB were related to dryness. The high-intensity UVB radiation is a complex abiotic stress factor that causes excessive light, heat, and dehydration to affect plant growth [42]. Severe plant dehydration will lead to changes in the structure of the plant photosynthetic system. The core gene of dehydration stress protein DSP-22 has long been found to participate in the protection of photosynthesis under plant drying stress [43]. Therefore, it was found that *Pohlia nutans* M211 formed a gene regulatory network with antioxidant, ion transport, and drought resistance as the core under the low-temperature and UVB conditions, respectively.

Plants can tolerate environmental changes by modifying the protein through AS mode under adverse conditions. The mutants lacking splicing elements or related components were less vulnerable to abiotic stresses [44]. According to KEGG analysis of AS genes, replication and repair, lipid metabolism, metabolism of cofactors and vitamins, metabolism of terpenoids and polyketides, amino acid metabolism, and environmental adaptation played important roles in *Pohlia nutans* M211 adaptation to low-temperature and intense UVB in Antarctica. As a result of the formation of oxygen free radicals caused by low-temperature and UV stress, the stability of plant genetic components is affected. AS genes of replication and repair can repair the damaged genetic material in time. In addition, the related AS genes of the metabolism of terpenoids and polyketides can produce more functional active substances to eliminate the free radicals produced in plants, protecting and repairing the damages caused by plant stress at the fastest speed. There 59 genes related to lipid metabolism were identified in *peanut* cold tolerance research, which indicates that lipid metabolism is an important strategy to cope with temperature stress [45]. Metabolic adaptation is the key pathway for the plant to resist abiotic stress because the accumulation of specific amino acids is related to the increase of plant tolerance under environmental conditions. For example, proline can increase the osmotic pressure of plant cells and is positively correlated with salt stress and low-temperature stress [46]. As a result, the key functional genes found along the pathways that are rich in AS genes may be useful for understanding how plants withstand stress.

Exploring the key PPI protein molecules and their interaction networks provides important clues for plant development regulation. The Antarctic environment is exceedingly severe, with low temperatures and intense radiation. HY5 regulates a large number of genes by directly combining with cis-regulatory elements, such as hormones, nutrients, abiotic stress (abscisic acid, salt, cold), and reactive oxygen species signaling pathway, which is one of the centers of the transcription network adapting to environmental stress [11]. In this study, the interaction network with HY5 as the core mainly included UVB, photosynthesis, secondary metabolites, temperature response, etc., and the functional genes were identified and discussed respectively. UVB radiation is the intrinsic part of sunlight and has important biological effects on the growth and development of plants. UVR8 is a UVB photoreceptor dissociated into monomers through tryptophan residues, and this monomer combines with COP1 to initiate signal transmission. COP1 is an inhibitor of photomorphogenesis, promoter of photosensitive pigment B reaction, and signal transducer mediated by UVB [47, 48]. The UVR8-COP1 complex recombines with the SPA protein and promotes transcription accumulation of downstream transcription factors (HY5, HYH and MYB12) to regulate the expression of downstream genes, including functional secondary metabolites flavonoid synthesis-related enzymes (CHS) and terpene synthesis-related enzymes (PDS and PSY) [49]. The secondary metabolites produced subsequently can absorb UVB and improve plant antioxidant levels. HY5 plays an important role in plant temperature morphogenesis except for regulating light morphogenesis. It can activate the pathway of DET1—COP1—HY5, PIF4 and other substances to exert their antifreeze activities [50]. B-box domain proteins 7 (BBX7) and 8 (BBX8) are direct targets of HY5 and actively regulate frost resistance by regulating the expression of a group of cold response genes, which are mainly independent of the C- repeat binding factor pathway [51]. To better withstand UVB stress and raise its level of frost tolerance, *Pohlia nutans* M211 upregulated the expression of UVR8 and HY5, while downregulating the expression of COP1. PHYB is a stable photoreceptor and is involved in the biosynthesis of photosynthetic pigments, chloroplast development, and many other processes [52]. The enhancement of plant resistance to UVB radiation via PHYB might be related to the increase of chlorophyll, carotenoid and flavonoid in leaves [53]. In addition, the CHS, HY5, and antioxidant enzymes APX1 and GPX were positively correlated PHYB [54]. PHYB regulates plant growth by sensing not only light signals but also environmental temperature signals. For example, studies have found that CBFs-PIF3-PHYB played an important regulatory role in modulating plant responses to low-temperature stress. Our findings that the expression level of at least one of the three PHYB copy genes was increased under low-temperature and UVB conditions, indicating the important position of PHYB in the stress resistance process. The increase of ABA content in plant cells is a signal for plants to sense and acclimate to the external environment [55]. LHCB is an apolipoprotein of the photosystem II light-harvesting complex that exhibits increased production of high-level ABA under stress conditions so that the low-temperature and UV light stress can make ABA increase rapidly in plants and enhance resistance. CHL catalyzes the terminal reaction in the biosynthesis pathway, causing the phytol or geraniol to combine with the C_17_ propionic acid of chlorophyll [56]. Increasing the expression of CHL in *Pohlia nutans* M211 to improve its photosynthetic capability is also one of the adaptation strategies to the Antarctic environment. TTL1 is related to osmotic stress and regulates the transcription levels of various dehydration-responsive genes (transcription factor DREB2A, ERD1, ERD3, and COR15a) and positive regulators of ABA signals in the presence of environmental stress [57]. The intense UVB environment is easy to cause plant dehydration and induces early dehydration reaction, therefore, high expression of TTL1 is helpful to improve the tolerance of plants to low-temperature and drought. Numerous research have demonstrated that increasing soluble sugars (fructose, sucrose and glucose) are beneficial to improving the cold resistance of plants [58]. Fructose-1, 6-bisphosphate (FBP) converts fructose-1,6- bisphosphate to glucose -6- phosphate and is involved in the Calvin cycle. Therefore, plants promote carbon metabolism by enhancing the activity of FBP at low temperatures [59]. In a word, PPI network interaction analysis with HY5 as the core discovered essential genes in photosynthesis, UVR8, secondary metabolites, and temperature response modules, laying a theoretical foundation for further investigation of the adaptation mechanism of *Pohlia nutans* M211.

## Conclusions

In this study, 43,101 full-length non-redundant sequences of *Pohlia nutans* M211 transcriptome were obtained by PacBio SMRT technique. The polar adaptation mechanisms of *Pohlia nutans* M211 mainly include an antioxidant system, secondary metabolites, photosynthesis, and gene repair through TF, KEGG, AS, WGCNA, and PPI analysis methods, combined with the results of the transcriptome, RT-qPCR, and biochemical detection kits.

## Materials and methods

### Plant materials

The moss *Pohlia nutans* M211 from the Antarctic was collected from the land near the Great Wall Station of China in Antarctica (62°13.233ʹS, 58°57.304ʹW) during the 33th Antarctic Expedition of China in 2017. The conditions of cultivation were performed on the soil media in an illumination incubator at 15 ± 1 °C with a relative humidity of 60%, under a white light density of 80 μmol photons m ^−2^ s ^−1^ with a diurnal cycle of 16 h light with 8 h dark.

### Temperature treatments

It is reported that the daytime temperature in the Antarctic continent ranges from -5 to 13 °C in summer [60], which is the main breeding and growth period of Antarctic bryophytes [61]. In addition, *Pohlia nutans* M211 have been cultured in an artificial climate box at 15 °C for rapid propagation since they were collected from the Antarctic, Therefore, the temperature of experiment was set at 0 °C, 7.5 °C and 15 °C after comprehensive consideration. The *Pohlia nutans* M211 seedlings were exposed to temperature levels (0 and 7.5 °C) at four-time points (2 h, 4 h, 8 h, 12 h and 24 h). The green gametophytes were cut off and frozen in liquid nitrogen immediately, and three biological replications were realized. The 15 °C was the temperature of the control treatment.

### UVB treatments

UVB lamps (280-320 nm, Huaqiang Nanjing, China) were used as the source of UVB irradiation [62], and the temperature during UVB radiation was set to 0 ℃ to simulate the Antarctic low-temperature environment. The UVB intensity of the Antarctic continent changes with the day sequence, and the UVB intensity varies from 0 to 160 µW/cm^2^ after detection [63]. The *Pohlia nutans* M211 seedlings were treated with UVB irradiance at UVB irradiation levels for 0 h (control), 2 h, 4 h, 8 h, 12 h and 24 h. The green gametophyte was frozen in liquid nitrogen immediately after cutting off and was analyzed in triplicate.

### Total RNA isolation

Total RNA was extracted using Trizol (Invitrogen, USA) by following the user manual. RNA degradation and purity (OD260/280) were measured using 1% agarose gel and Nanodrop (IMPLEN, Germany). Accurate quantification of RNA concentration and accurate detection of RNA integrity was performed using Qubit (Invitrogen Life Technologies, USA) and Agilent 2100 (Agilent Technologies, USA), respectively.

### Library preparation and sequencing

The iso-seq library was prepared according to the isoform sequencing protocol (iso-seq), which the cleantech smarter cDNA synthesis kit and the blue pippin size selection system protocol were described by specific biosciences (PN 100–092-800–03).

### Data analysis

The raw data were processed using the SMRTlink 5.0 software, and circular consensus sequence (CCS) was generated from subread BAM files (min_length 200, max_drop_fraction 0.8, no_polish TRUE, min_zscore -9999, min_passes 1, min_predicted_accuracy 0.8). FLNC (full-length nonchimera) and nFL (Non-Full-Length) sequences were identified by detecting whether CCS contained 5'-primer, 3'-primer, and poly-A. The FLNC sequences of the same transcript were clustered with a hierarchical n*log(n) algorithm to obtain a consensus sequence. Finally, the acquired full-length sequence was subjected to polish, and the polished consensus sequence was used for subsequent analysis. The polish consensus sequence achieved in the preceding stage was corrected using the second-generation data, and then the CD-HIT software (cd-hit v4.6.8) was then used for redundancy and as reference sequences. The clean reads from each sample acquired by Illumina sequencing were then compared to reference sequences.

### Functional and structural annotations of transcriptome genes

CDS (Coding sequence) is a sequence encoding a protein product, and ANGEL software (ANGEL V2.4, https://github.com/PacificBiosciences/ANGEL) was used for CDS prediction analysis. iTAK software (iTAK: 1.7a, https://github.com/kentnf/iTAK/) was used to predict plant transcription factors (TF). Simple sequence repeats (SSR) was analyzed with MISA software (version 1.0, default parameter, http://pgrc.ipk-gatersleben.de/misa/misa.html), and the minimum repeats for each unit size were as follows: 1–10, 2–6, 3–5, 4–5, 5–5, 6–5. LncRNAs (long non-coding RNA) do not encode proteins, which was predicted by CNCI (CNCI v2, https://github.com/www-bioinfo-org/CNCI), PLEK(PLEK v1.2, https://sourceforge.net/projects/plek/), CPC2 (CPC2 v0.1, http://cpc2.cbi.pku.edu.cn/download.php) and Pfam (PfamScan V1.6, https://www.ebi.ac.uk/seqdb/confluence/display/THD/PfamScan) database, and then analyze the final lncRNA.

Gene functions were annotated using the following databases: NCBI non-redundant protein sequences (Nr) (https://www.ncbi.nlm.nih.gov/protein/), NCBI nucleotide sequences (Nt) (https://www.ncbi.nlm.nih.gov/nucleotide/), Protein family (Pfam) (http://pfam.sanger.ac.uk/), Clusters of Orthologous Groups of proteins and euKaryotic Ortholog Groups (KOG/COG) (http://www.ncbi.nlm.nih.gov/COG/), Swiss-Prot (http://www.ebi.ac.uk/uniprot/), Kyoto Encyclopedia of Genes and Genomes (KEGG) (http://www.genome.jp/kegg/).

### Quantification of gene expression level and differential expression analysis

The expression level of genes was analyzed by RSEM software (RSEM V1.3.0, http://deweylab.github.io/RSEM/) for each sample, and gene expression levels were estimated by fragments per kilobase of transcript per million fragments mapped (FPKM). Differential expression analysis of two groups was performed by using DESeq2 (DEseq v1.10.1, http://www.bioconductor.org/packages/release/bioc/html/DESeq.html), which used a model based on the negative binomial distribution. The *p* values were adjusted using Benjamini and Hochberg’s approach for controlling the false discovery rate (FDR). Genes with FDR-adjusted *p*-value < 0.05 found by DESeq were assigned as differentially expressed genes [64], and |log_2_ (Fold Change)|> 1 and *p* < 0.05 as the threshold for significantly differential expression.

### Gene enrichment analysis

Kyoto Encyclopedia of Genes and Genomes (KEGG) was a database for systematic analysis of gene function and genomic information, which helped researchers study genes and expression information as a whole network. It was assigned to each gene based on the KOBAS software (KOBAS v3.0, http://kobas.cbi.pku.edu.cn/download.php).

### Selection of suitable reference genes and RT-qPCR

The genes expressed in all samples with FPKM > 0, which were compared with the comparison combinations and filtered out differential genes [65]. The top 20 genes were then selected in Norm Finder software to enter the reference gene screen based on their expression levels. The two genes with the lowest stability value (60S and eIF4A) were used as reference genes for RT-qPCR by the normalization method.

The RT-qPCR was employed to validate candidate DEGs associated with cold tolerance and UVB resistance, which was performed on TransStart Green qPCR SuperMix (AQ101-01, TransGen Biotech Co., Ltd, Beijing, China) for 40 cycles (95 °C for 5 s; 58 °C for 10 s; 72 °C for 40 s). The primers were designed by Sangon Biotech Co., Ltd. (Shanghai, China) and were listed in Table S5. The data was further analyzed using the comparative Ct (2^−∆∆CT^) method [62].

### Determination of antioxidant, secondary metabolites, and chlorophyll levels

Samples (gametophytes) for antioxidant level determination were taken at 1, 2, 4, 8, 12 h at low-temperature and UVB treatment, with the same temperature and UVB intensity as for transcriptome sampling. The samples of secondary metabolites (except carotenoids) and chlorophyll levels were sampled at 1, 3, 7, 21, and 35 day, and the samples of carotenoid levels were sampled at 21, 35 days under low-temperature and UVB treatment, respectively. The detection kit of POD (peroxidase), SOD (superoxide dismutase), MDA (malondialdehyde), H_2_O_2_, flavonoid, polyphenol, soluble sugar, and chlorophyll were purchased from Nanjing Jiancheng Bioengineering Institute (Nanjing, China), and the above experimental operations were performed according to the instructions repeated three times [66].

### Statistical analysis

The statistical analyses employed software of SPSS 22.0 (SPSS Inc., Chicago, USA) by using one-way analysis of variance (ANOVA) with Tukey. Each of the groups’ values were expressed as the means ± standard deviation (SD), *p* < 0.05 and *p* < 0.01 were considered to reflect a statistically significant difference and a highly significant difference, respectively.

### Supplementary Information

**Additional file 1. **

**Additional file 2.**

**Additional file 3.**

**Additional file 4.**

**Additional file 5.**

**Additional file 6.**

**Additional file 7: Supplemental Table 1.** Summary of reads from third-generation sequencing. **Supplemental Table 2.** Summary of Illumina sequencing. (15℃, 7.5℃ and 0℃ represented the different temperatures of the moss in the temperature treatment respectively, and 40μW/cm2,80μW/cm2 and 160μW/cm2 represented the different UVB intensities of the moss at 15℃). **Supplemental Table 3.** The number of up-regulated and down-regulated expressions of TFs under different conditions. (7.5 °C vs15 °C and 0 °C vs15 °C represented the comparison between moss at 7.5 °C and 0 °C and 15 °C, respectively. 40 μW/cm2 vs 0 μW/cm2, 80 μW/cm2 vs 0 μW/cm2, and 160 μW/cm2 vs 0 μW/cm2 represented the UVB radiation intensity of moss at 40 μW/cm2, 80 μW/cm2 and 160 μW/cm2 compared with 0 μW/cm2, respectively). **Supplemental Table 4.** The description of core genes in modules MEbrown, MEturqoise and MEpurple. **Supplemental Table 5.** The list of primers. **Supplemental Table 6.** Under different conditions, the functions and expression levels of key genes in different modules of the PPI network.

**Additional file 8: Figure S1.** The transcription factor families screening. (The a is the top 30 with the largest number of transcripts analysis, different transcription factor families are abscissa; number of TF is ordinate. The c and d are the up-regulated and down-regulated expression changes of different transcription factors at 0°C and 7.5°C compared with 15°C. The c, e and f are the up-regulated and down-regulated expression changes of different transcription factors at 160 μW/cm^2^, 40 μW/cm^2^ and 80 μW/cm^2^ compared with 0 μW/cm^2^. T0, T7.5 and T15 represent 0℃, 7.5℃ and 15℃, respectively. UV40, UV80 and UV160 represent 40 μW/cm^2^, 80 μW/cm^2^ and 160 μW/cm^2^, respectively. T0 also represents 0 μW/cm^2^, which will not be repeated below, the red bars represent upregulation and blue bars represent down regulation).

**Additional file 9: Figure S2.** The length distribution map of lncRNA (a) and graph of gene expression correlation analysis (b).

**Additional file 10: Figure S3.** The volcanic variations under different conditions.

**Additional file 11: Figure S4.** The differential gene Venn diagrams for different conditions.

**Additional file 12: Figure S5.** The soft threshold determination of gene co-expression networks.

**Additional file 13: Figure S6.** The expression levels of all genes and corresponding ME in different modules.

## Data Availability

The raw sequence data generated in this study have been deposited in the National Center for Biotechnology Information under accession number PRJNA889080 for *Pohlia nutans* M211 (BioSample accession: SAMN31232186). This BioProject submission and BioSample database will be released on 2023–12-12 or upon publication, whichever is first.

## Abbreviations

ADK: Adenylate kinase
AS: Alternative splicing
COP1: Constitutively photomorphogenic 1
CHS: Chalcone synthase
CHL: Chlorophyll synthase
DEGs: Differentially expressed genes
FBP: Fructose-1,6-bisphosphate
GSH: Glutathione
GSSG: Oxidized glutathione
HY5: Hlongated hypocotyl 5
LHCB: Light-harvesting chlorophyll a/b binding protein
MDA: Malondialdehyde
PSBD: Photosystem II protein D2
PSBN: Photosystem II N protein
PSII: Photosystem II protein
PDS: Phytoene desaturase
PSY: Phytoene synthase
POD: Peroxidase, PHYB, Phytochrome B
SOD: Superoxide dismutase
SMRT: Single-molecular real-time
TTL1: Atricoptide-repeat thioredoxin-like1
PPI: Protein–protein interaction networks
UVR8: UV resistance locus 8
WGCNA: Weighted gene co-expression network analysis

## References

[1] Roads E, Longton RE, Convey P (2014). Millennial timescale regeneration in a moss from Antarctica. Current biology : CB.

[2] Lindo Z, Gonzalez A (2010). The Bryosphere: An Integral and Influential Component of the Earth's Biosphere. Ecosystems.

[3] Wardle DA, Jonsson M, Bansal S, Bardgett RD, Gundale MJ, Metcalfe DB (2015). Linking vegetation change, carbon sequestration and biodiversity. J Ecol.

[4] Kyung DK, Young YM, Hee SJ, Jae KY, Young KM, Suk-Ha L (2015). Underlying genetic variation in the response of cultivated and wild soybean to enhanced ultraviolet-B radiation. Euphytica.

[5] Hossaini R, Chipperfield MP, Montzka SA, Leeson AA, Dhomse SS, Pyle JA (2017). The increasing threat to stratospheric ozone from dichloromethane. Nat Commun.

[6] Fabón G, Monforte L, Tomás R, Martinez-Abaigar J (2013). Cell Compartmentation of UV-Absorbing Compounds in Two Aquatic Mosses Under Enhanced UV-B. Cryptogam Bryol.

[7] Fabón G, Monforte L, Tomás-Las-Heras R, Núñez-Olivera E, Martínez-Abaigar J (2012). Dynamic response of UV-absorbing compounds, quantum yield and the xanthophyll cycle to diel changes in UV-B and photosynthetic radiations in an aquatic liverwort. J Plant Physiol.

[8] Soriano G, Cloix C, Heilmann M, Núñez-Olivera E, Martínez-Abaigar J, Jenkins GI (2018). Evolutionary conservation of structure and function of the UVR8 photoreceptor from the liverwort Marchantia polymorpha and the moss Physcomitrella patens. New Phytol.

[9] Wolf L, Rizzini L, Stracke R, Rensing USA (2010). The Molecular and Physiological Responses of Physcomitrella patens to Ultraviolet-B Radiation. Plant Physiol.

[10] Fernández MB, Tossi V, Lamattina L, Cassia R (2016). A comprehensive phylogeny reveals functional conservation of the UV-B photoreceptor UVR8 from Green Algae to Higher Plants. Front Plant Sci.

[11] Gangappa SN, Botto JF (2016). The multifaceted roles of HY5 in plant growth and development. Molecular Plant.

[12] Oliver MJ, Mishler T (2000). The evolution of vegetative desiccation tolerance in land plants. Plant Ecol.

[13] Perera-Castro AV, Waterman MJ, Turnbull JD, Ashcroft MB, McKinley E, Watling JR, Bramley-Alves J, Casanova-Katny A, Zuniga G, Flexas J (2020). It is hot in the sun: antarctic mosses have high temperature optima for photosynthesis despite cold climate. Front Plant Sci.

[14] Ma Y, Zhang Y, Jiang L, Shao HJAJoB. Roles of plant soluble sugars and their responses to plant cold stress. Afr J Biotechnol. 2009;8(10):2004–10.

[15] Yu J, Cang J, Lu Q, Fan B, Xu Q, Li W, Wang X (2020). ABA enhanced cold tolerance of wheat 'dn1' via increasing ROS scavenging system. Plant Signal Behav.

[16] Clayton WA, Albert NW, Thrimawithana AH, McGhie TK, Deroles SC, Schwinn KE, Warren BA, McLachlan ARG, Bowman JL, Jordan BR (2018). UVR8-mediated induction of flavonoid biosynthesis for UVB tolerance is conserved between the liverwort Marchantia polymorpha and flowering plants. Plant J.

[17] Catalá R, Medina J, Salinas J (2011). Integration of low temperature and light signaling during cold acclimation response in Arabidopsis. Proc Natl Acad Sci USA.

[18] Gangappa SN, Botto JF (2016). The Multifaceted Roles of HY5 in Plant Growth and Development. Mol Plant.

[19] Yang L, Cao H, Zhang X, Gui L, Chen Q, Qian G, Xiao J, Li Z. Genome-Wide Identification and Expression Analysis of Tomato ADK Gene Family during Development and Stress. Multidisciplinary Digital Publishing Institute. 2021;22(14):7708. 10.3390/ijms22147708 PMC8305589 34299327

[20] Khudyakova AY, Kreslavski VD, Shirshikova GN, Zharmukhamedov SK, Kosobryukhov AA, Allakhverdiev SI (2017). Resistance of Arabidopsis thaliana L. photosynthetic apparatus to UV-B is reduced by deficit of phytochromes B and A. J Photochem Photobiol B Biol.

[21] Manish S, Renu M, Himanshu S, Srikanth T, Chary K, Rao BJ, Rajagopal S (2012). UVI31+ Is a DNA Endonuclease That Dynamically Localizes to Chloroplast Pyrenoids in C. reinhardtii. Plos One.

[22] Shuang L, Gongxue J, Haiping T, Yujun W, Binye L, Qien Y (2022). Effects of hypobaric hypoxia on spermatogenesis and the expression of small RNA in mice. Acta Theriologica Sinica.

[23] Feng H, Li S, Xue L, An L, Wang X (2007). The interactive effects of enhanced UV-B radiation and soil drought on spring wheat. S Afr J Bot.

[24] Shang X, Ying C, Ma L (2017). Alternative splicing in plant genes: a means of regulating the environmental fitness of plants. Int J Mol Sci.

[25] Gao X, Wang Q, Xiang X, Zhu Y, Wang J, Wang W, Larkins BA, Wu Y (2020). Carotenoids modulate kernel texture in maize by influencing amyloplast envelope integrity. Nat Commun.

[26] Xie Z, Nolan TM, Jiang H, Yin Y (2019). AP2/ERF Transcription Factor Regulatory Networks in Hormone and Abiotic Stress Responses in Arabidopsis. Front Plant Sci.

[27] Rushton DL, Tripathi P, Rabara RC, Lin J, Ringler P, Boken AK, Langum TJ, Smidt L, Boomsma DD, Emme NJ (2012). WRKY transcription factors: key components in abscisic acid signalling. Plant Biotechnol J.

[28] Ding R, Che X, Shen Z, Zhang Y (2021). Metabolome and transcriptome profiling provide insights into green apple peel reveals light- and UV-B-responsive pathway in anthocyanins accumulation. BMC Plant Biol.

[29] Han G, Lu C, Guo J, Qiao Z, Sui N, Qiu N, Wang B (2020). C2H2 Zinc Finger Proteins: Master Regulators of Abiotic Stress Responses in Plants. Front Plant Sci.

[30] Gong P, Zhang J, Li H, Yang C, Zhang C, Zhang X, Khurram Z, Zhang Y, Wang T, Fei Z (2010). Transcriptional profiles of drought-responsive genes in modulating transcription signal transduction, and biochemical pathways in tomato. J Exp Bot.

[31] Zhou S, Shu W, Boone B, Levy S (2007). Microarray analysis of genes affected by salt stress in tomato. Afr J Environ Technol.

[32] Schwarz C, Bohne AV, Wang F, Cejudo FJ, Nickelsen J (2012). An intermolecular disulfide-based light switch for chloroplast psbD gene expression in Chlamydomonas reinhardtii. Plant J Cell Mol Biol.

[33] Torabi S, Umate P, Manavski N, Plöchinger M, Kleinknecht L, Bogireddi H, Herrmann RG, Wanner G, Schröder WP, Meurer J (2014). PsbN is required for assembly of the photosystem II reaction center in Nicotiana tabacum. Plant Cell.

[34] Kim SH, Kim M, Lee JK, Min JK, Sang DP (1997). Identification and expression of uvi31+, a UV-inducible gene from Schizosaccharomyces pombe. Environ Mol Mutagen.

[35] Kataria S, Jajoo A, Guruprasad KN (2014). Impact of increasing Ultraviolet-B (UV-B) radiation on photosynthetic processes. J Photochem Photobiol, B.

[36] Yang SH, Wang LJ, Li SH, Duan W, Loescher W, Liang ZC (2007). The effects of UV-B radiation on photosynthesis in relation to Photosystem II photochemistry, thermal dissipation and antioxidant defenses in winter wheat (Triticum aestivum L.) seedlings at different growth temperatures. Functional plant biology : FPB.

[37] Schulz E, Tohge T, Winkler JB, Albert A, Sch Ffner AR, Fernie AR, Zuther E, Hincha DK (2021). Natural Variation among Arabidopsis Accessions in the Regulation of Flavonoid Metabolism and Stress Gene Expression by Combined UV Radiation and Cold. Plant Cell Physiol.

[38] Masuda HP, Nakabashi M, Morgante PG, Kajihara D, Sluys M (2020). Evidence for sub-functionalization of tandemly duplicated XPB nucleotide excision repair genes in Arabidopsis thaliana. Gene.

[39] Zhou RI, Zhao YH, Yang RY, Zuo JC, Zhou BB (2015). Analysis of the different physiological responses of Leymus mollis( Trin. ) Hara leaves and roots to sand burial on the coast of Yantai China. Acta Ecologica Sinica.

[40] Srivastava P (2016). Provitamin A Carotenoids Reduce Consequences of Radiation Stress Apoptosis: Its Implications in Cancer Radiotherapy.

[41] Yuan P, Yang T, Poovaiah BW (2018). Calcium Signaling-Mediated Plant Response to Cold Stress. Int J Mol Sci.

[42] Min YY, Kim MY, Ha J, Lee T, Kim KD, Lee SH (2019). QTL Analysis of Resistance to High-Intensity UV-B Irradiation in Soybean (Glycine max [L.] Merr.). Int J Mol Sci.

[43] Bartels D, Hanke C, Schneider K, Michel D, Salamini F (1992). A desiccation-related Elip-like gene from the resurrection plant Craterostigma plantagineum is regulated by light and ABA. EMBO J.

[44] Punzo P, Grillo S, Batelli G (2020). Alternative splicing in plant abiotic stress responses. Biochem Soc Trans.

[45] Zhong C, Jiang C, Ren J, Dong J, Yu H (2020). An advanced lipid metabolism system revealed by transcriptomic and lipidomic analyses plays a central role in peanut cold tolerance. Front Plant Sci.

[46] Hildebrandt TM (2018). Synthesis versus degradation: directions of amino acid metabolism during Arabidopsis abiotic stress response. Plant Mol Biol.

[47] CL, Deng XW. COP1 - from plant photomorphogenesis to mammalian tumorigenesis. TR Cell Biol 2005, 15(11):618–625. 10.1016/j.tcb.2005.09.007 16198569

[48] Attila Oravecz AB, Zoltán Máté, Agnieszka Brzezinska, Jean Molinier, Edward J. Oakeley, va dám, Eberhard Schfer, Ferenc Nagy,, Ulm R. CONSTITUTIVELY PHOTOMORPHOGENIC1 Is Required for the UV-B Response in Arabidopsis. Plant Cell 2006;18(8):1975–1990. 10.1105/tpc.105.040097 PMC1533968 16829591

[49] Liu CC, Chi C, Jin LJ, Zhu J, Yu JQ, Zhou YH (2018). The bZip transscription factor HY5 mediates CRY1a -induced anthocyanin biosynthesis in tomato. Plant, Cell Environ.

[50] Delker C, Sonntag L, James GV, Janitza P, Ibañez C, Ziermann H, Peterson T, Denk K, Mull S, Ziegler JR (2014). The DET1-COP1-HY5 Pathway Constitutes a Multipurpose Signaling Module Regulating Plant Photomorphogenesis and Thermomorphogenesis. Cell Reports.

[51] Youping L, Yiting S, Minze L, Diyi F, Shifeng W, Jigang L, Zhizhong G, Hongtao L, Shuhua Y (2021). The CRY2–COP1–HY5–BBX7/8 module regulates blue light-dependent cold acclimation in Arabidopsis. Plant Cell.

[52] Coe J, Kupitz C, Basu S, Conrad CE, Fromme P (2015). Crystallization of Photosystem II for Time-Resolved Structural Studies Using an X-ray Free Electron Laser. Methods Enzymol.

[53] Joshi PN, Pradhan MK, Biswal B (2008). Interaction of Light Absorbed by Phytochrome and UV-B Radiation in Modulating the Composition and Function of Carotenoid in Photosynthetic Apparatus of Wheat Leaves: Role of UV-A Photoreceptor. Res J Biotechnol.

[54] Kreslavski VD, Strokina VV, Khudyakova AY, Shirshikova GN, Allakhverdiev SI (2021). Effect of high-intensity light and UV-B on photosynthetic activity and the expression of certain light-responsive genes in A thaliana phyA and phyB mutants. Biochim Biophys Acta Bioenerge.

[55] Yu F, Cao X, Liu G, Wang Q, Xie Q (2020). ESCRT-I Component VPS23A Is Targeted by E3 Ubiquitin Ligase XBAT35 for Proteasome-Mediated Degradation in Modulating ABA Signaling. Mol Plant.

[56] Proctor MS, Pazderník M, Jackson PJ, Piln J, Martin EC, Dickman MJ, Canniffe DP, Johnson MP, Hunter CN, Sobotka R (2020). Xanthophyll carotenoids stabilise the association of cyanobacterial chlorophyll synthase with the LHC-like protein HliD. Biochem J.

[57] Abel R, Schapire AL, Bressan RA, Harfouche AL, Hasegawa PM, Victoriano V, Botella MA (2018). The Arabidopsis tetratricopeptide repeat-containing protein TTL1 is required for osmotic stress responses and abscisic acid sensitivity. Plant Physiol.

[58] Liang G, Ma Z, Lu S, Ma W, Feng L, Mao J, Chen B (2022). Temperature-phase transcriptomics reveals that hormones and sugars in the phloem of grape participate in tolerance during cold acclimation. Plant Cell Rep.

[59] Stitt M, Hurry V. A plant for all seasons: alterations in photosynthetic carbon metabolism during cold acclimation in Arabidopsis. 2002, 5(3):199-206 10.1016/s1369-5266(02)00258-3 11960736

[60] Convey P, Coulson SJ, Worland MR, Sjöblom A (2018). The importance of understanding annual and shorter-term temperature patterns and variation in the surface levels of polar soils for terrestrial biota. Polar Biol.

[61] Waterman MJ, Nugraha AS, Hendra R, Ball GE, Robinson SA, Keller PA (2017). Antarctic Moss Biflavonoids Show High Antioxidant and Ultraviolet-Screening Activity. J Nat Prod.

[62] An M, Qu C, Miao J, Sha Z (2021). A Class II CPD Photolyase and a 6–4 Photolyase with Photorepair Activity from the Antarctic Moss Pohlia nutans M211. Photochem Photobiol.

[63] Zheng X, Cheng H (2020). Comparison of solar UV radiation measurements at Zhongshan station, Antarctica. J Appl Meteorol Sci.

[64] Storey JD, Tibshirani R (2003). Statistical significance for genomewide studies. Proc Natl Acad Sci USA.

[65] Daniel R, Wu Q, Williams V, Clark G, Guruli G (2017). A Panel of MicroRNAs as Diagnostic Biomarkers for the Identification of Prostate Cancer. Int J Mol Sci.

[66] Zhao Y, He Y, Wang X, Qu C, Miao J (2022). Proline metabolism regulation in Spartina alterniflora and SaP5CS2 gene positively regulates salt stress tolerance in transgenic Arabidopsis thaliana. J Plant Interact.

