# Supplementary material for: Extreme environmental adaptation mechanisms of Antarctic bryophytes are mainly the activation of antioxidants, secondary metabolites and photosynthetic pathways
Source: BMC Plant Biol. 2023 Aug 22;23:399. doi: 10.1186/s12870-023-04366-w (PMC10464054; doi:10.1186/s12870-023-04366-w)
Supplement: Supplementary file 7 — Additional file 7: Supplemental Table 1. Summary of reads from third-generation sequencing. Supplemental Table 2. Summary of Illumina sequencing. (15℃, 7.5℃ and 0℃ represented the different temperatures of the moss in the temperature treatment respectively, and 40μW/cm2,80μW/cm2 and 160μW/cm2 represented the different UVB intensities of the moss at 15℃). Supplemental Table 3. The number of up-regulated and down-regulated expressions of TFs under different conditions. (7.5 °C vs15 °C and 0 °C vs15 °C represented the comparison between moss at 7.5 °C and 0 °C and 15 °C, respectively. 40 μW/cm2 vs 0 μW/cm2, 80 μW/cm2 vs 0 μW/cm2, and 160 μW/cm2 vs 0 μW/cm2 represented the UVB radiation intensity of moss at 40 μW/cm2, 80 μW/cm2 and 160 μW/cm2 compared with 0 μW/cm2, respectively). Supplemental Table 4. The description of core genes in modules MEbrown, MEturqoise and MEpurple. Supplemental Table 5. The list of primers. Supplemental Table 6. Under different conditions, the functions and expression levels of key genes in different modules of the PPI network. [file 12870_2023_4366_MOESM7_ESM.docx]

**Supplemental Table 1** Summary of reads from third-generation sequencing

| Item | Number | Mean length | N50 |
| --- | --- | --- | --- |
| Polymerase Reads | 1074872 | 53947 | 111535 |
| Subreads | 29661756 | 1881 | 2247 |
| CCS | 1074872 | 2409 | 2584 |
| FLNC | 831751 | 2204 | 2409 |
| Polished consensus | 78059 | 2217 | 2431 |
| The correct polished consensus | 78059 | 2216 | 2430 |
| The transcripts after redundancy removal | 43101 | 2268 | 2487 |

**Supplemental Table 2** Summary of Illumina sequencing. (15℃, 7.5℃ and 0℃ represented the different temperatures of the moss in the temperature treatment respectively, and 40μW/cm^2^,80μW/cm^2^ and 160μW/cm^2^ represented the different UVB intensities of the moss at 15℃)

| Sample | Raw_reads | Clean_reads | Clean_bases | GC |
| --- | --- | --- | --- | --- |
| 15 ℃_1 | 47377322 | 45942768 | 6.89G | 53.03 |
| 15 ℃_2 | 43277136 | 42032948 | 6.3G | 53.04 |
| 15 ℃_3 | 44451098 | 43125130 | 6.47G | 52.9 |
| 7.5 ℃_1 | 43775672 | 42298568 | 6.34G | 52.78 |
| 7.5 ℃_2 | 43699524 | 42460552 | 6.37G | 52.8 |
| 7.5 ℃_3 | 46088756 | 44682602 | 6.7G | 52.9 |
| 0 ℃_1 | 46934154 | 45393624 | 6.81G | 53.05 |
| 0 ℃_2 | 41840762 | 40498464 | 6.07G | 53.19 |
| 0 ℃_3 | 45747858 | 44353022 | 6.65G | 53.16 |
| 160 μW/cm^2^_1 | 43689636 | 42119406 | 6.32G | 53 |
| 160 μW/cm^2^_2 | 44657424 | 42645320 | 6.4G | 52.82 |
| 160 μW/cm^2^_3 | 46000128 | 44334410 | 6.65G | 52.92 |
| 80 μW/cm^2^_1 | 48317400 | 46552186 | 6.98G | 52.56 |
| 80 μW/cm^2^_2 | 45867078 | 44226604 | 6.63G | 52.81 |
| 80 μW/cm^2^_3 | 42392620 | 40783048 | 6.12G | 52.99 |
| 40 μW/cm^2^_1 | 47984406 | 45751696 | 6.86G | 53.04 |
| 40 μW/cm^2^_2 | 42287140 | 40685916 | 6.1G | 52.87 |
| 40 μW/cm^2^_3 | 46675290 | 42586336 | 6.39G | 53.01 |

**Supplemental Table 3** The number of up-regulated and down-regulated expressions of TFs under different conditions. (7.5 °C vs15 °C and 0 °C vs15 °C represented the comparison between moss at 7.5 °C and 0 °C and 15 °C, respectively. 40 μW/cm^2^ vs 0 μW/cm^2^, 80 μW/cm^2^ vs 0 μW/cm^2^, and 160 μW/cm^2^ vs 0 μW/cm^2^ represented the UVB radiation intensity of moss at 40 μW/cm^2^, 80 μW/cm^2^ and 160 μW/cm^2^ compared with 0 μW/cm^2^, respectively)

| Groups | AP2/ERF | | WRKY | | bHLH | | bZIP | | MYB | | NAC | | C2H2 | |
| --- | --- | --- | --- | --- | --- | --- | --- | --- | --- | --- | --- | --- | --- | --- |
|  | UP | Down | UP | Down | UP | Down | UP | Down | UP | Down | UP | Down | UP | Down |
| 7.5 ℃ vs15℃ | 59 | 1 | 21 | 2 | 22 | 7 | 39 | 5 | 17 | 3 | 22 | 1 | 12 | 2 |
| 0℃ vs15℃ | 56 | 19 | 1 | 8 | 13 | 10 | 23 | 2 | 1 | 10 | 10 | 11 | 13 | 5 |
| 40 μW/cm^2^ vs  0 μW/cm^2^ | 42 | 5 | 8 | 0 | 5 | 8 | 3 | 16 | 5 | 3 | 8 | 3 | 1 | 1 |
| 80μW/cm^2^ vs  0 μW/cm^2^ | 67 | 3 | 4 | 0 | 3 | 10 | 10 | 3 | 3 | 2 | 20 | 1 | 0 | 2 |
| 160μW/cm^2^vs 0μW/cm^2^ | 44 | 3 | 4 | 0 | 4 | 5 | 4 | 5 | 4 | 3 | 8 | 2 | 0 | 2 |

**Supplemental Table 4 The description of core genes in modules MEbrown, MEturqoise and MEpurple**

|  | Gene ID | Accession number | GO/KEGG | Function |
| --- | --- | --- | --- | --- |
| MEbrown | transcript_HQ_TX_pn_transcript8932/f2p0/3315 | XP_001784706.1 | oxidation-reduction process | oxidoreductase activity |
|  | transcript_HQ_TX_pn_transcript70207/f9p0/1165 | XP_001776520.1 | oxidation-reduction process//chloride transport | FMN binding//pyridoxamine-phosphate oxidase activity |
|  | transcript_HQ_TX_pn_transcript56996/f3p0/1686 | XP_001772136.1 | oxidation-reduction process | oxidoreductase activity |
|  | transcript_HQ_TX_pn_transcript65791/f2p0/1346 | XP_001781305.1 | protein folding | cytoplasm |
|  | transcript_HQ_TX_pn_transcript10532/f3p0/3193 | ABF66648.1 | oxidation-reduction process | oxidoreductase activity |
|  | transcript_HQ_TX_pn_transcript74256/f3p0/896 | XP_001785912.1 |  |  |
|  | transcript_HQ_TX_pn_transcript73764/f2p0/946 | XP_001768609.1 |  |  |
|  | transcript_HQ_TX_pn_transcript73017/f4p0/1004 | XP_001752782.1 | translation | intracellular//ribosome |
|  | transcript_HQ_TX_pn_transcript55440/f89p0/1661 | XP_001761873.1 | regulation of transcription | DNA binding//nucleotide binding |
|  | transcript_HQ_TX_pn_transcript31714/f13p0/2281 | XP_001753540.1 |  |  |
| MEturqoise | transcript_HQ_TX_pn_transcript53666/f3p0/1767 | XP_001775039.1 |  |  |
|  | transcript_HQ_TX_pn_transcript71631/f5p0/1107 | XP_001768152.1 |  |  |
|  | transcript_HQ_TX_pn_transcript72429/f2p0/1057 |  |  |  |
|  | transcript_HQ_TX_pn_transcript42738/f2p0/2049 | XP_001781524.1 |  |  |
|  | transcript_HQ_TX_pn_transcript34347/f3p0/2255 | XP_001756965.1 | ion transport | ion channel activity |
|  | transcript_HQ_TX_pn_transcript49934/f13p0/1845 | XP_001765303.1 |  |  |
|  | transcript_HQ_TX_pn_transcript61446/f3p0/1571 | XP_001773033.1 | positive regulation of transcription elongation from RNA polymerase II promoter | DNA binding |
|  | transcript_HQ_TX_pn_transcript39044/f2p0/2125 | XP_001760231.1 |  | protein binding |
|  | transcript_HQ_TX_pn_transcript36819/f2p0/2193 | XP_001782565.1 |  |  |
|  | transcript_HQ_TX_pn_transcript60539/f4p0/1544 | XP_001755688.1 |  |  |
| MEpurple | transcript_HQ_TX_pn_transcript75456/f2p0/770 |  |  |  |
|  | transcript_HQ_TX_pn_transcript77629/f2p0/285 |  |  |  |
|  | transcript_HQ_TX_pn_transcript61939/f5p0/1551 | XP_001766493.1 | Putative transferase | Transcription factor, component of CCR4 transcriptional complex |
|  | transcript_HQ_TX_pn_transcript63125/f3p0/1491 | XP_001753766.1 |  | Desiccation stress protein DSP-22 |
|  | transcript_HQ_TX_pn_transcript33393/f4p0/2250 | XP_001763031.1 |  |  |
|  | transcript_HQ_TX_pn_transcript72186/f4p0/1058 | XP_001753653.1 |  | Desiccation stress protein DSP-22 |
|  | transcript_HQ_TX_pn_transcript41570/f18p0/2012 | XP_001779959.1 |  |  |
|  | transcript_HQ_TX_pn_transcript63039/f3p0/1502 | XP_001753766.1 |  | Desiccation stress protein DSP-22 |
|  | transcript_HQ_TX_pn_transcript71073/f5p0/1142 | XP_001753653.1 |  | Desiccation stress protein DSP-22 |
|  | transcript_HQ_TX_pn_transcript76586/f2p0/607 |  | response to stress |  |

**Supplemental Table 5 The list of primers**

| Gene name | Forward primer | Reverse primer |
| --- | --- | --- |
| UVR8 | ATATCTGGTGGTTGGCGGCATTC | TCCACATCCCACTTGACCAAACTTG |
| HY5 | CTTCTTCTCCGCCCTCATCAAAGC | CTGGAACCCTCCTAACATCGCAATC |
| COP1 | ACATCAAGTCACATATCGCCAGCAG | CCTTCACCTTGCCGTCATCACTC |
| PSY | AGAGAACAGTGTGCGTGAATTGGAC | TGTGAAGTTGTCGTAGTCGTTGGC |
| PDS | CCGCTAAGTACGTGGCTGATTCTG | CCTTGTCCTTCCATGCTGCTACC |
| CHS | GTACATGGAGCCGTCGCTGAAC | ATGTGCGTGATGTCGGACTTGC |
| CHL | GCTCCGTCAAGTCGTTCTCATCTG | CGCTACCGTTGAATTGTTGGCTTC |
| FBP | CAGAGCACATCCACGACAGATCAC | ACTTGGCAGATTCAGCGGCATAC |
| 60s (reference gene) | GCCAGACCCGAAGATCCGTATTTAC | TCTCCTTCTCCCACGACACCAAG |
| eIF4 (reference gene) | GTGAGTTCCGCTCTGGTTCGTC | GGGTTGGCAGGTCGTAGTTGATG |

**Supplemental Table 6 Under different conditions, the functions and expression levels of key genes in different modules of the PPI network**

| Gene ID | Predict protein | 40 μW/cm^2^ **vs** 0μW/cm^2^ | | 80 μW/cm^2^ **vs** 0μW/cm^2^ | | 160 μW/cm^2^ **vs** 0μW/cm^2^ | | 7.5 ℃ vs 15 ℃ | | 0℃ vs 15℃ | |
| --- | --- | --- | --- | --- | --- | --- | --- | --- | --- | --- | --- |
|  |  | log2FC | Padj | log2FC | Padj | log2FC | Padj | log2FC | Padj | log2FC | Padj |
| transcript_HQ_TX_pn_transcript9330/f3p0/3292  transcript_HQ_TX_pn_transcript9183/f12p0/3157 * | HY5 | -0.838  2.858 | 0.019  0.000 | -0.315  3.303 | 0.645  0.000 | -0.848  2.149 | 0.185  0.579 | 1.572  2.683 | 0.006  0.053 | 3.8333  1.948 | 0.000  0.053 |
| transcript_HQ_TX_pn_transcript9380/f3p0/3285  transcript_HQ_TX_pn_transcript9048/f2p0/3274  transcript_HQ_TX_pn_transcript5231/f2p0/3687 *  transcript_HQ_TX_pn_transcript16505/f4p0/2842 | COP1 | -1.577  -1.586  -1.305  -0.802 | 0.001  0.000  0.000  0.005 | -0.472  -0.697  -0.957  0.092 | 0.575  0.054  0.012  0.987 | -0.996  -1.196  -0.937  -0.436 | 0.210  0.022  0.149  0.628 | 1.358  1.713  0.034  Inf | 0.005  0.000  0.095  0.017 | 2.267  3.309  0.941  Inf | 0.000  0.000  0.002  0.000 |
| transcript_HQ_TX_pn_transcript31525/f4p0/2360  transcript_HQ_TX_pn_transcript39482/f2p0/2131  transcript_HQ_TX_pn_transcript9815/f2p0/3135 * | UVR8 | -1.054  -2.124  Inf | 0.017  0.000  0.000 | -2.059  -1.311  -0.249 | 0.000  0.000  0.812 | -1.739  -2.634  -1.262 | 0.008  0.000  0.028 | 4.456  1.303  1.838 | 0.000  0.166  0.175 | 6.154  4.101  5.176 | 0.000  0.000  0.000 |
| transcript_HQ_TX_pn_transcript1671/f20p0/4346  transcript_HQ_TX_pn_transcript1522/f8p0/4459  transcript_HQ_TX_pn_transcript1582/f3p0/4428 | PHYB | -1.434  -1.540  7.030 | 0.000  0.000  0.016 | -1.427  -1.592  9.222 | 0.000  0.000  0.006 | -1.349  -1.548  5.668 | 0.001  0.000  0.707 | 1.853  0.524  - | 0.000  0.073  - | 3.639  2.434  -4.864 | 0.000  0.000  0.462 |
| transcript_HQ_TX_pn_transcript73075/f4p0/1003  transcript_HQ_TX_pn_transcript72294/f25p0/1030  transcript_HQ_TX_pn_transcript71348/f2p0/1128 | LHCB2 | Inf  -0.761  -0.578 | 0.000  0.001  0.006 | -0.282  -0.556  -0.328 | 0.897  0.035  0.235 | -0.713  -0.459  -0.322 | 0.366  0.573  0.758 | 0.783  -0.548  -0.462 | 0.674  0.012  0.217 | -0.184  0.880  0.683 | 0.566  0.000  0.000 |
| transcript_HQ_TX_pn_transcript52184/f4p0/1814  transcript_HQ_TX_pn_transcript51177/f2p0/1844 * | PSY | -0.751  -0.558 | 0.000  0.003 | -0.663  -0.487 | 0.000  0.014 | -0.898  -0.321 | 0.027  0.744 | 0.804  0.707 | 0.000  0.000 | 2.198  1.940 | 0.000  0.000 |
| transcript_HQ_TX_pn_transcript24911/f7p0/2497  transcript_HQ_TX_pn_transcript33673/f10p0/2235 * | PDS | -0.941  -1.316 | 0.025  0.000 | -1.453  -1.077 | 0.000  0.002 | -1.312  -1.417 | 0.025  0.008 | 1.823  0.665 | 0.000  0.008 | 1.752  0.813 | 0.000  0.007 |
| transcript_HQ_TX_pn_transcript60077/f2p0/1605  transcript_HQ_TX_pn_transcript54823/f15p0/1741 *  transcript_HQ_TX_pn_transcript53047/f339p0/1720  transcript_HQ_TX_pn_transcript54219/f78p0/1683  transcript_HQ_TX_pn_transcript50759/f65p0/1833  transcript_HQ_TX_pn_transcript46928/f3p0/1858  transcript_HQ_TX_pn_transcript53980/f7p0/1776 | CHS | -1.059  -1.161  -1.054  -1.034  -0.892  -0.766  -1.738 | 0.005  0.000  0.004  0.009  0.002  0.003  0.002 | -1.574  -1.828  -1.456  -1.768  -1.646  -1.185  -2.188 | 0.000  0.000  0.000  0.000  0.000  0.000  0.000 | -0.819  -0.503  -0.483  -0.650  -1.044  0.076  -1.359 | 0.108  0.506  0.537  0.2840.0.037  1  0.077 | 1.964  2.155  1.765  2.718  2.531  1.957  2.922 | 0.000  0.000  0.000  0.000  0.000  0.000  0.000 | 1.510  1.585  1.270  1.891  2.279  1.496  2.742 | 0.000  0.000  0.000  0.000  0.000  0.000  0.000 |
| transcript_HQ_TX_pn_transcript56330/f43p0/1660 *  transcript_HQ_TX_pn_transcript10583/f3p0/3122 | ChL | -0.739  Inf | 0.046  0.002 | -0.077  Inf | 0.985  0.000 | -0.398  Inf | 0.779  0.000 | -1.086  -1.241 | 0.000  0.813 | 0.162  - | 0.673  - |
| transcript_HQ_TX_pn_transcript1439/f2p0/4442 | TTL1 | Inf | 0.027 | Inf | 0.141 | Inf | 0.888 | -3.479 | 0.382 | - | - |
| transcript_HQ_TX_pn_transcript66209/f36p0/1345 *  transcript_HQ_TX_pn_transcript65241/f7p0/1422  transcript_HQ_TX_pn_transcript57231/f2p0/1690  transcript_HQ_TX_pn_transcript64805/f5p0/1417 | FBPase | -0.997  -0.978  -1.606  -1.113 | 0.000  0.001  0.000  0.000 | -0.823  -1.487  -1.343  -0.727 | 0.000  0.000  0.007  0.007 | -0.841  -1.719  -1.306  -0.561 | 0.061  0.000  0.058  0.445 | 0.3545  0.551  1.499  0.284 | 0.127  0.064  0.948  0.285 | 1.548  1.549  2.240  1.028 | 0.000  0.000  0.000  0.000 |
