# Supplementary figures and images for: Extreme environmental adaptation mechanisms of Antarctic bryophytes are mainly the activation of antioxidants, secondary metabolites and photosynthetic pathways
Source: BMC Plant Biol. 2023 Aug 22;23:399. doi: 10.1186/s12870-023-04366-w (PMC10464054; doi:10.1186/s12870-023-04366-w)

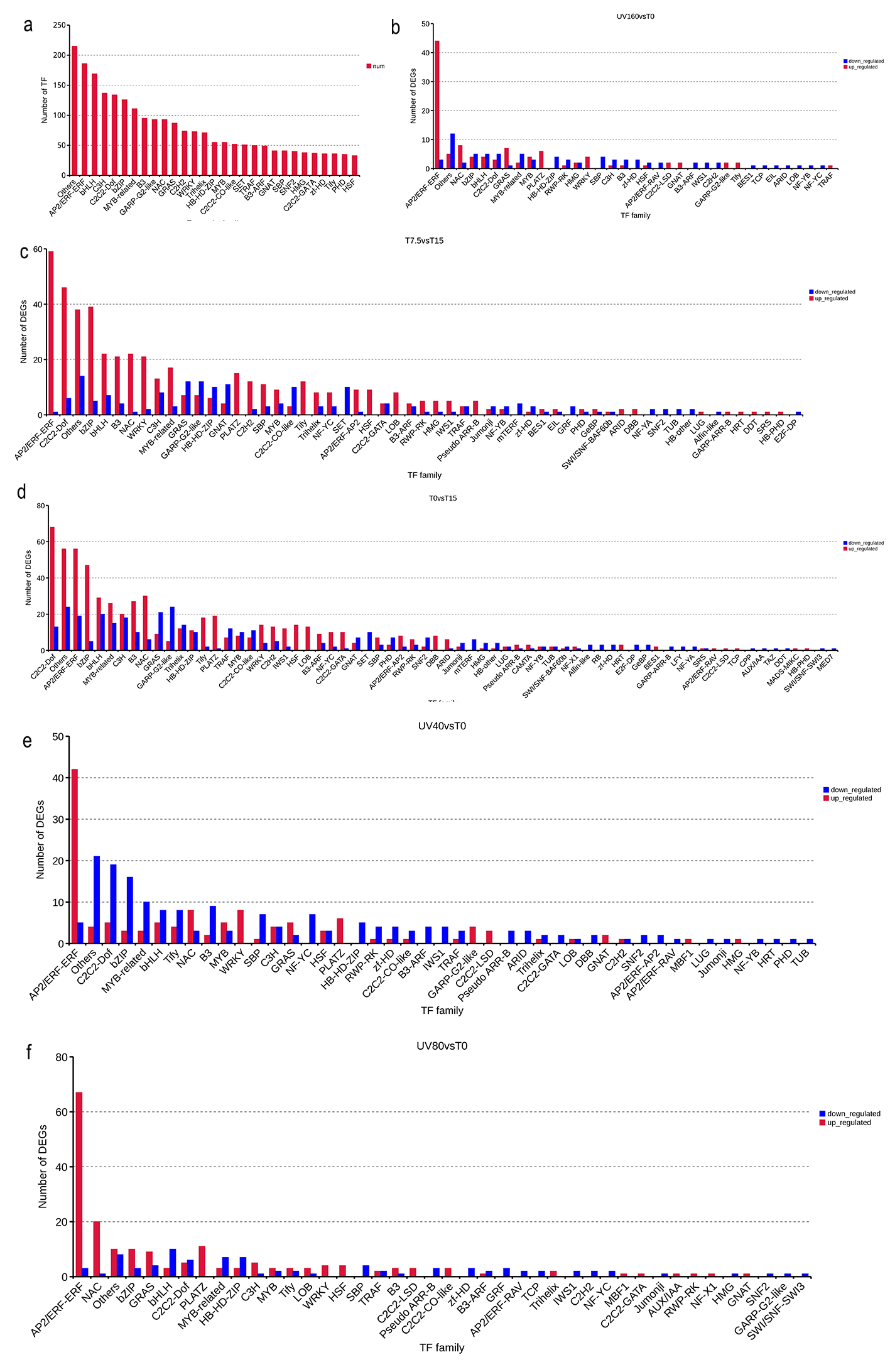

Supplement: Supplementary file 8 — Additional file 8: Figure S1. The transcription factor families screening. (The a is the top 30 with the largest number of transcripts analysis, different transcription factor families are abscissa; number of TF is ordinate. The c and d are the up-regulated and down-regulated expression changes of different transcription factors at 0°C and 7.5°C compared with 15°C. The c, e and f are the up-regulated and down-regulated expression changes of different transcription factors at 160 μW/cm2, 40 μW/cm2 and 80 μW/cm2 compared with 0 μW/cm2. T0, T7.5 and T15 represent 0℃, 7.5℃ and 15℃, respectively. UV40, UV80 and UV160 represent 40 μW/cm2, 80 μW/cm2 and 160 μW/cm2, respectively. T0 also represents 0 μW/cm2, which will not be repeated below, the red bars represent upregulation and blue bars represent down regulation). [file 12870_2023_4366_MOESM8_ESM.tif]

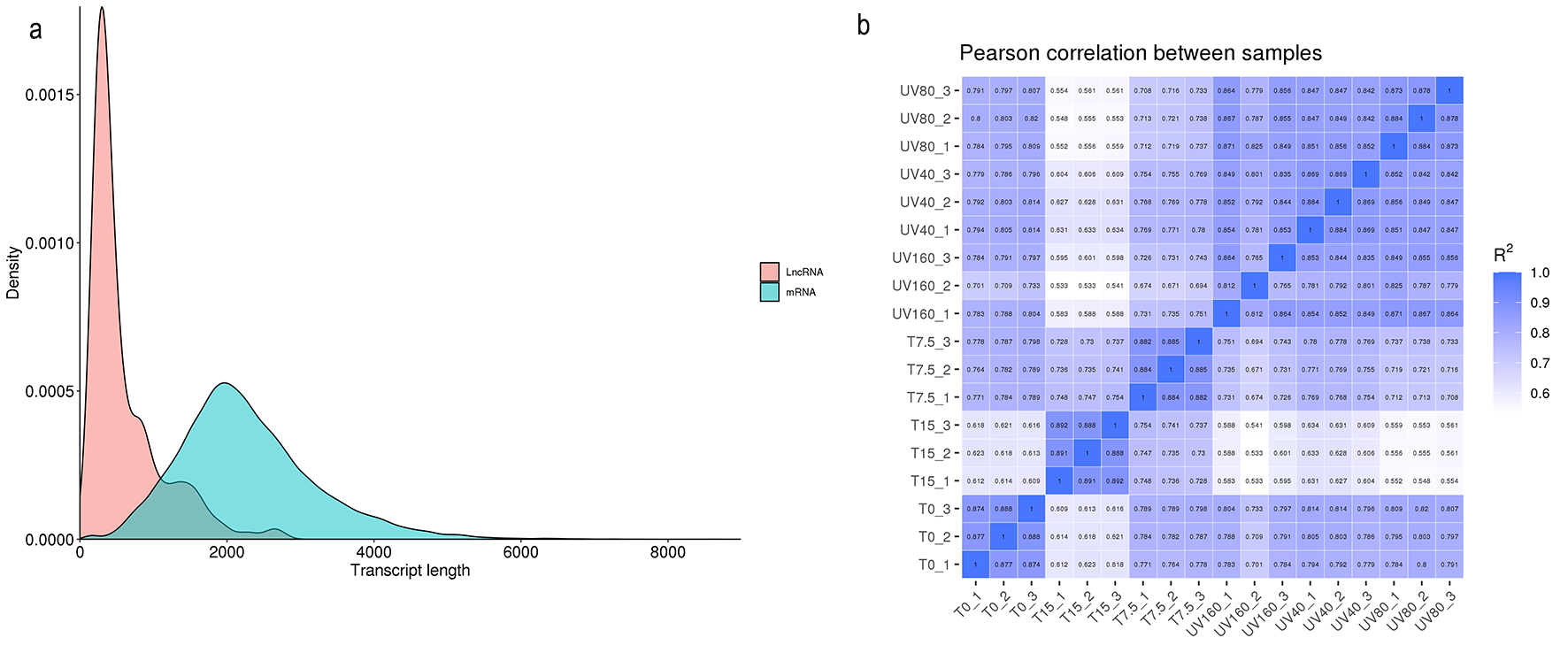

Supplement: Supplementary file 9 — Additional file 9: Figure S2. The length distribution map of lncRNA (a) and graph of gene expression correlation analysis (b). [file 12870_2023_4366_MOESM9_ESM.tif]

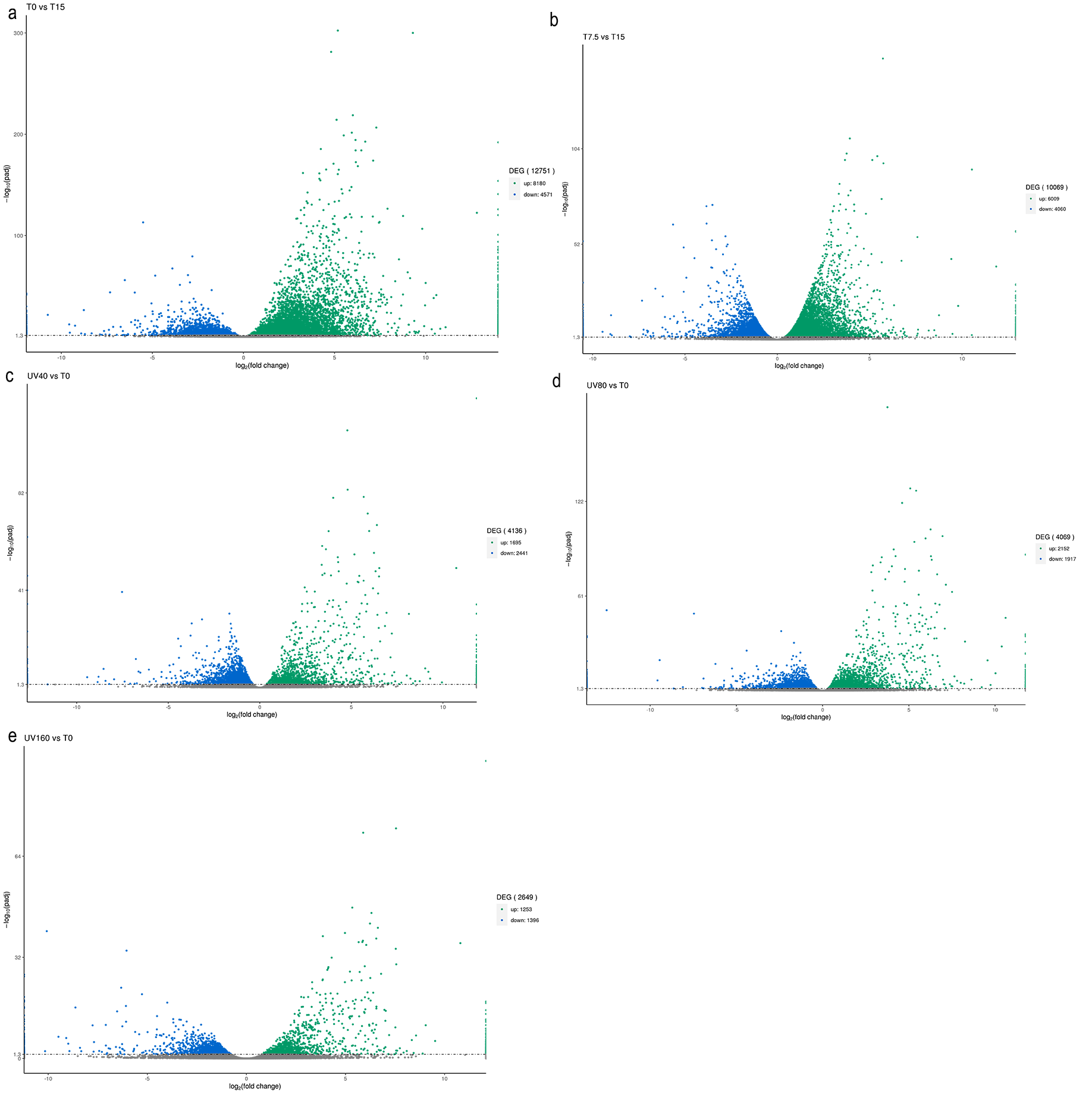

Supplement: Supplementary file 10 — Additional file 10: Figure S3. The volcanic variations under different conditions. [file 12870_2023_4366_MOESM10_ESM.tif]

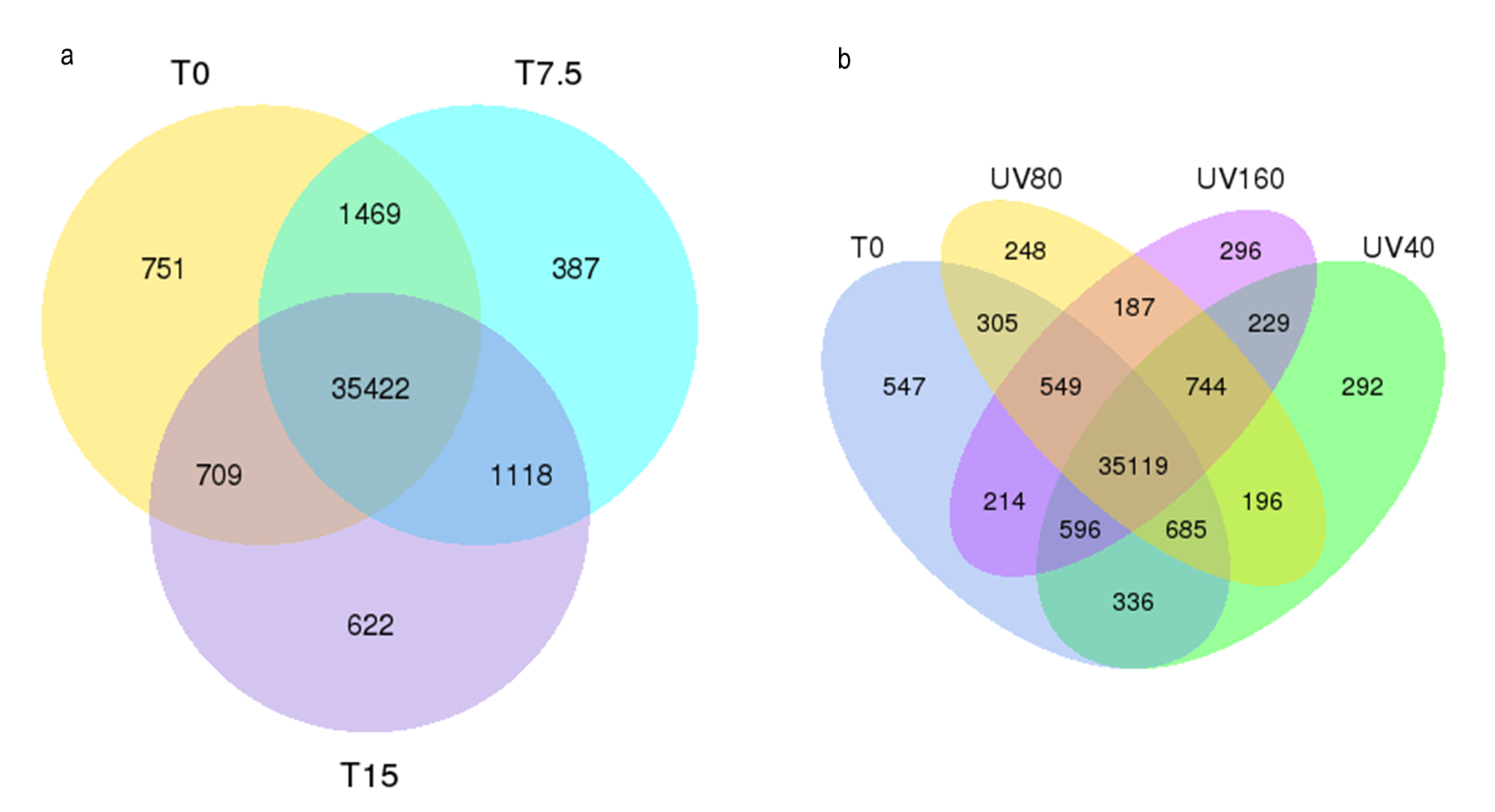

Supplement: Supplementary file 11 — Additional file 11: Figure S4. The differential gene Venn diagrams for different conditions. [file 12870_2023_4366_MOESM11_ESM.tif]

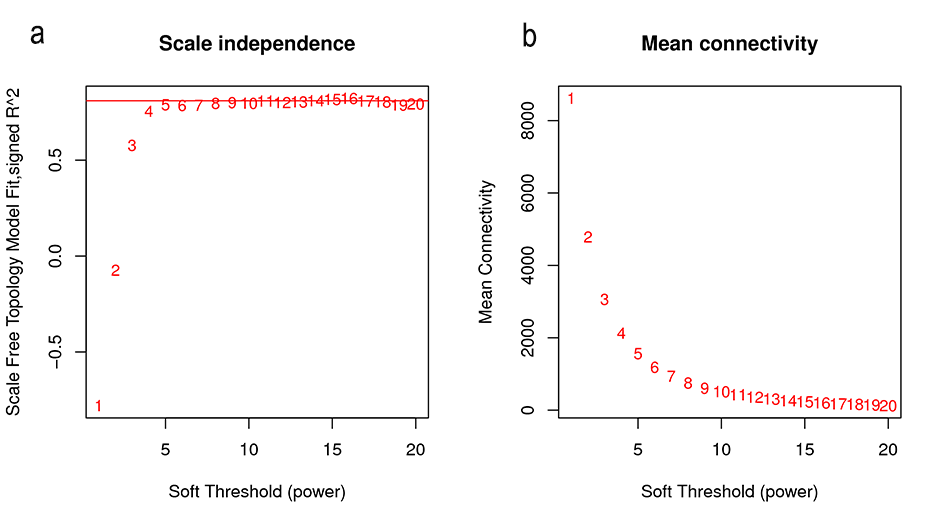

Supplement: Supplementary file 12 — Additional file 12: Figure S5. The soft threshold determination of gene co-expression networks. [file 12870_2023_4366_MOESM12_ESM.tif]

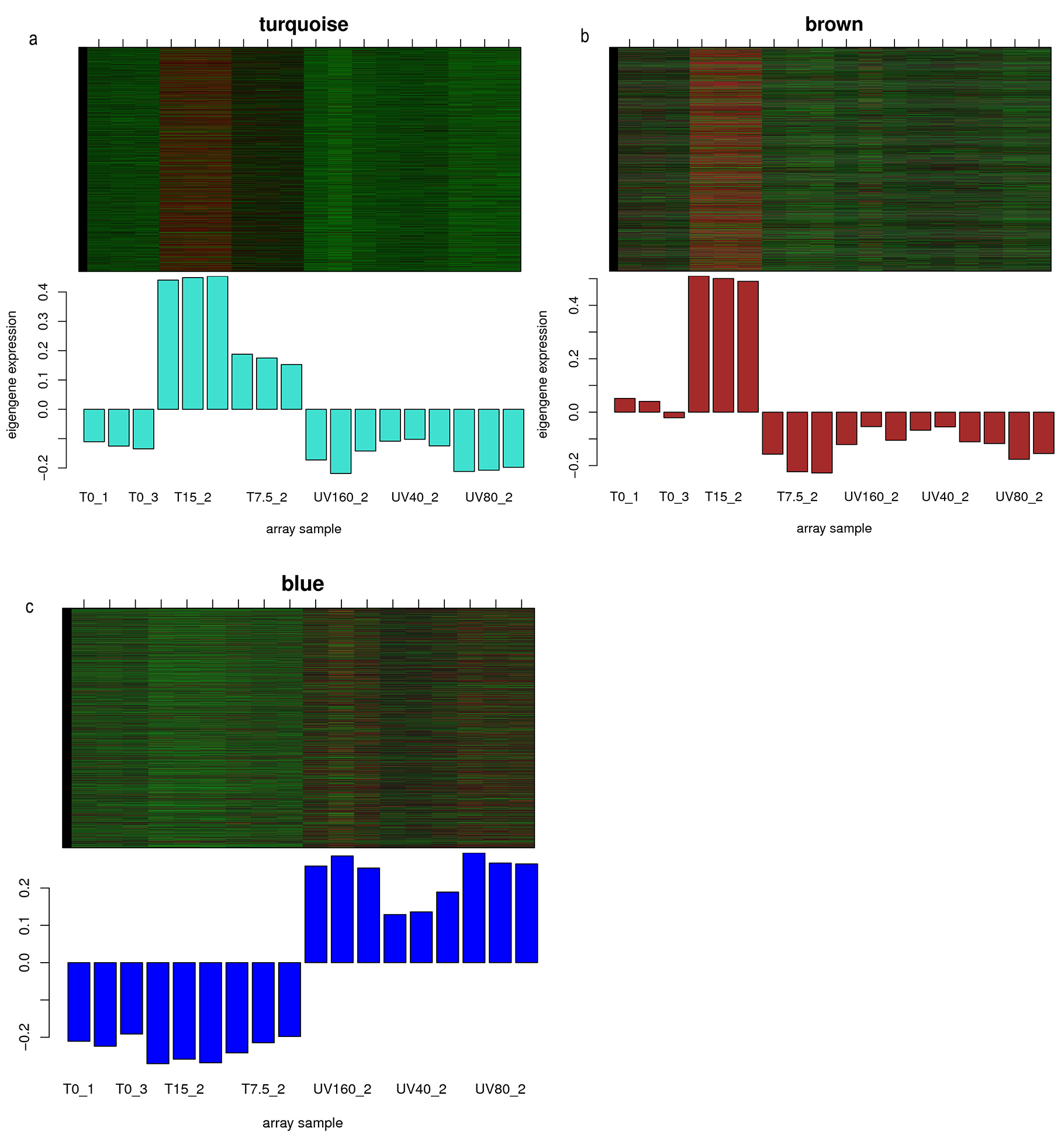

Supplement: Supplementary file 13 — Additional file 13: Figure S6. The expression levels of all genes and corresponding ME in different modules. [file 12870_2023_4366_MOESM13_ESM.tif]
